# Inertial Sensor Technology for Elite Swimming Performance Analysis: A Systematic Review

**DOI:** 10.3390/s16010018

**Published:** 2015-12-25

**Authors:** Robert Mooney, Gavin Corley, Alan Godfrey, Leo R Quinlan, Gearóid ÓLaighin

**Affiliations:** 1 Electrical & Electronic Engineering, School of Engineering & Informatics, NUI Galway, University Road, Galway, Ireland; r.mooney4@nuigalway.ie (R.M.); gavin.corley@nuigalway.ie (G.C.); 2 Bioelectronics Research Cluster, National Centre for Biomedical Engineering Science, NUI Galway, University Road, Galway, Ireland; 3 Institute for Neuroscience, Newcastle University, Newcastle upon Tyne, Tyne and Wear NE1 7RU, UK; alan.godfrey@newcastle.ac.uk; 4 Physiology, School of Medicine, NUI Galway, University Road, Galway, Ireland; 5 CÚRAM (SFI Centre for Research in Medical Devices), NUI Galway, University Road, Galway, Ireland; gearoid.olaighin@nuigalway.ie

**Keywords:** swimming, inertial sensor, accelerometer, gyroscope, kinematics, stroke analysis, MEMS, biomechanics, performance analysis

## Abstract

Technical evaluation of swimming performance is an essential factor of elite athletic preparation. Novel methods of analysis, incorporating body worn inertial sensors (*i.e.*, Microelectromechanical systems, or MEMS, accelerometers and gyroscopes), have received much attention recently from both research and commercial communities as an alternative to video-based approaches. This technology may allow for improved analysis of stroke mechanics, race performance and energy expenditure, as well as real-time feedback to the coach, potentially enabling more efficient, competitive and quantitative coaching. The aim of this paper is to provide a systematic review of the literature related to the use of inertial sensors for the technical analysis of swimming performance. This paper focuses on providing an evaluation of the accuracy of different feature detection algorithms described in the literature for the analysis of different phases of swimming, specifically starts, turns and free-swimming. The consequences associated with different sensor attachment locations are also considered for both single and multiple sensor configurations. Additional information such as this should help practitioners to select the most appropriate systems and methods for extracting the key performance related parameters that are important to them for analysing their swimmers’ performance and may serve to inform both applied and research practices.

## 1. Introduction

Elite swimming is highly competitive, with world class athletes constantly challenging themselves against their rivals and tiny margins deciding the outcome of races. Consequently, swimmers and coaches continually strive for methods and strategies to optimise performance. A fundamental aspect of this preparation involves regular, quantifiable data measurement to assess skill acquisition and technical development.

**Figure 1:**
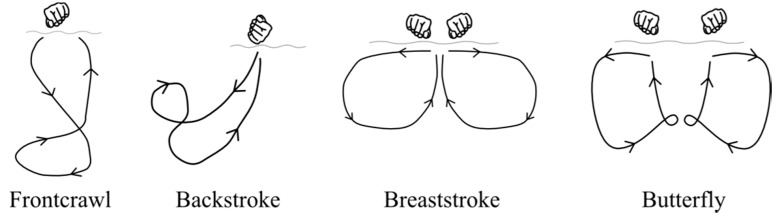
Representation of typical arm actions during swimming, highlighting the characteristic patterns of movement and sweeps of the arms for each of the four competitive strokes. Adapted from Maglischo [1].

Swimming is characterised by a sequence of coordinated actions of the trunk and limbs, in a repeated, synchronous pattern. Arm action during each of the four competitive swimming strokes comprises specific phases. It is typical to define these phases according to the various sweeps of the arms, which are specific to each stroke (Figure 1). For example downsweep; insweep; and upsweep movements are completed during frontcrawl [1]. Important kinematic variables such as velocity and acceleration fluctuate greatly throughout each phase, both for specific body segments and the body as a whole. Techniques for accurately determining this valuable information can therefore be used for quantitative biomechanical analysis and to inform the coaching process.

**Figure 2:**
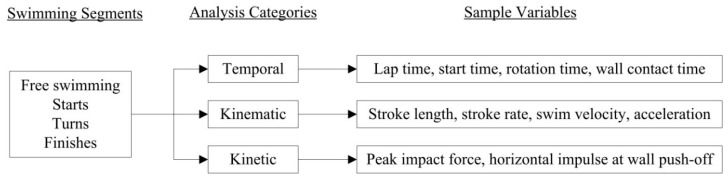
Swimming can be broken down into different segments to facilitate technical analysis and different categories of performance related variables can be selected for measurement.

Competitive swimming can be broken down into specific segments to facilitate such analysis (Figure 2). Starts are typically defined as the duration from the starting buzzer until the swimmer reaches the 15 m mark. Turns are defined according to coaches’ requirements and involve varying distances on approach to and leaving the wall after each lap. For example, competition analysis performed at major international competitions have defined this segment from 5 m before the wall to 5 m after the wall [2]. Finishes involve the final few meters (typically 5 m) before the wall is touched at the end of the race. Finally, free swimming is the term given to describe the regular swimming strokes performed during each lap that occurs outside of the other segments. During each of these race segments, different categories of analysis are appropriate and can take place through the measurement of temporal, kinematic and kinetic variables. Examples of swimming variables related to each category are provided in Figure 2 and may be examined with various methods.

Predominant methods for extracting this quantitative information are video-based [3]. Images from cameras positioned above and/or below the water allow for the entire swimming stroke to be captured, yielding vast amounts of information such as velocity profiling [4] or joint angular kinematic analysis [5]. Video capture in aquatic environments has inherent disadvantages however, such as parallax error, hidden or obscured body segments and water turbulence. Moreover, the digitization and data analysis process associated with video analysis is labour intensive and time consuming, thus reducing its effectiveness as a feedback tool [6,7]. A recent survey of swimming coaches also found that although quantitative analysis is perceived to be important, the time consuming nature of the process is limiting its application in practice [8].

Recent advances in the development of microelectromechanical systems (MEMS); wearable technologies and waterproofed coatings facilitate a potentially new approach to swimming coaching. These advances may allow for the development of new kinematic swim sensor technology which facilitates improved analysis of stroke mechanics, race performance and evaluation of exercise intensity thus enabling more efficient, competitive and quantitative coaching. This has led some to suggest that this technology may offer significant advantages over traditional video based approaches [9].

A number of authors have developed the use of MEMS systems for measuring key performance related parameters in swimming [10,11,12]. An important consideration in this ongoing development work is feature extraction. However, a thorough evaluation of different feature detection algorithms described in the literature and the consequences associated with different sensor attachment locations is warranted and has been cited by Magalhaes, *et al.* [13] as an important gap in the literature. By way of example, various algorithms have been described for measuring the same parameter, such as velocity, and often using devices placed at different locations on the body; but the relative merits of these approaches has not yet been examined in detail. This has led to substantial ambiguity on the optimal system design; most suitable algorithms for a given parameter of interest and best means of applying kinematic swim sensor technologies, significantly limiting their potential in applied settings.

Indeed it was suggested by Magalhaes, Vannozzi, Gatta and Fantozzi [13] that there has been poor uptake of this technology by coaches for these reasons, with research evidence also supporting this claim [8]. The aim of this systematic review is to address these gaps in the literature and to provide further depth of understanding of this growing area of research. Additional information such as this should help practitioners to select the most appropriate systems and methods for extracting the key performance related parameters that are important to them for analysing their swimmers’ performance and may serve to inform both applied and research practices.

## 2. Methods

### 2.1. Review Questions

A systematic review of the literature into the application of inertial sensor technology for the analysis of swimming performance was conducted in an attempt to address the following review questions: (1) What signal processing methods have been utilised to measure parameters for the analysis of the different swimming race segments, including free-swimming, starts and turns? (2) What is the current functionality and performance of commercially available swimming sensor devices? (3) What are the implications for the placement of these sensors at different body sites on device functionality? (4) What technical specifications are required for the optimum design of kinematic swim sensor technologies?

### 2.2. Article Selection

Article selection was based on a systematic search for publications following the Prisma guidelines [14] of the following scientific databases: Embase; European Patent Office; IEEE Xplore; ISI Web of Knowledge; PatentScope (World Intellectual Property Organisation); PubMed; Science Direct; Scopus; SPORT Discus and the United States Patent and Trademark Office. These databases were chosen as the most relevant sources of information related to the areas of engineering; sports science and sports technology. All publications from January 2000 to May 2015 were included in the search. The keyword string used for the search was “(swimming OR frontcrawl OR freestyle OR backstroke OR backcrawl OR breaststroke OR butterfly) AND (accelerometer OR gyroscope OR inertial sensor OR IMU (Inertial Measurement Unit) OR MEMS OR acceleration OR angular velocity)”. In this context, IMU and MEMS are commonly used acronyms for Inertial Measurement Unit and Micro Electro Mechanical Systems, respectively. The inclusion criteria were that the publication: (i) was written in English; (ii) appeared in a peer-reviewed academic source or patent; (iii) was related to the analysis of human competitive swimming. Exclusion criteria included: (i) animal studies and (ii) publications not directly related to the topics outlined in the review questions.

## 3. Results

**Figure 3:**
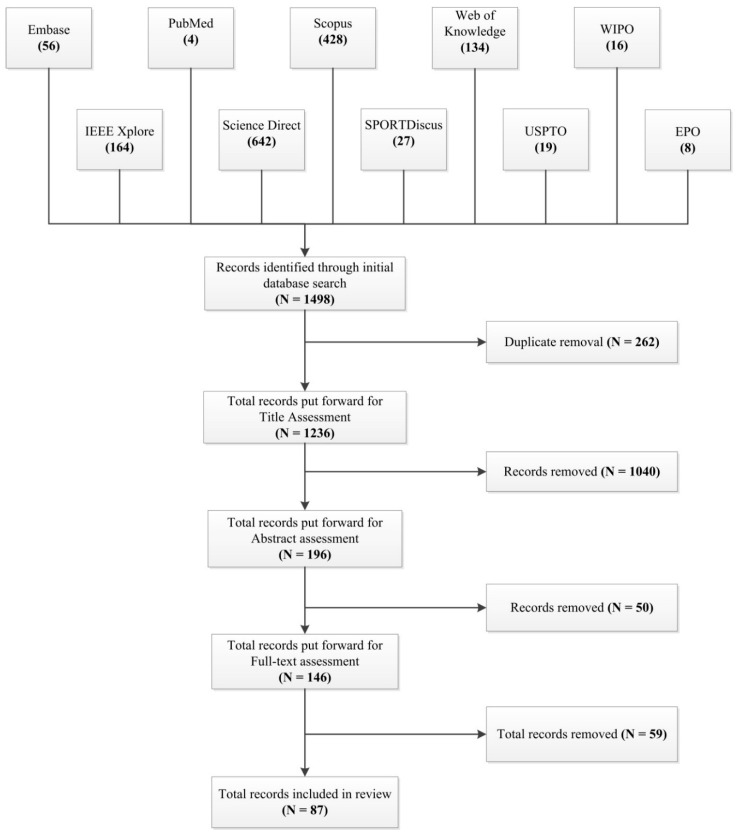
Systematic review search strategy and results.

**Figure 4:**
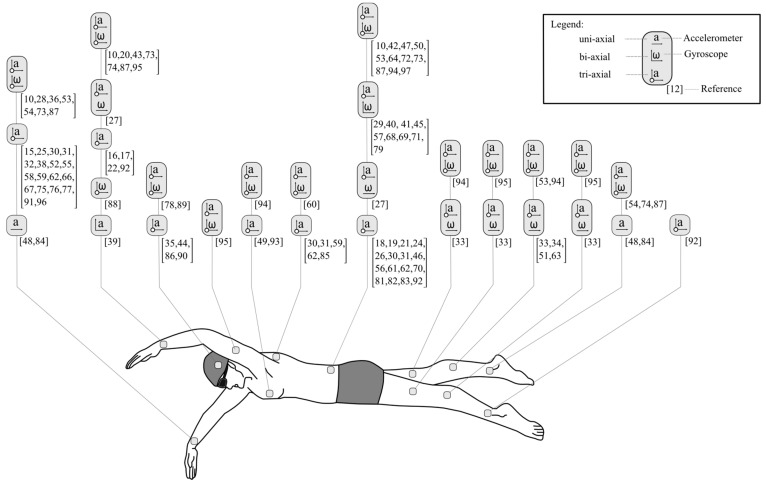
Locations and specifications of different inertial sensor units used in previous swimming related studies. Studies have used devices in both single and multiple sensor configurations. The most popular locations are the lower back and wrist/lower arm and the most prevalent sensor specifications incorporate a tri-axial accelerometer and tri-axial gyroscope.

The process flowchart detailing the results of the database search and article selection is provided in Figure 3. The initial search yielded 1498 results. Duplicates were removed and the title and abstract of each publication was reviewed and evaluated based on the relevance to the systematic review questions. The final number of publications included for this review was 87. Table 1 provides a summary of the publications selected and includes information related to the participants involved in these studies; the swimming strokes examined; the sensor output variables that were extracted; the phase of swimming that the variables are relevant to and the validation method used to verify the results of the study. Figure 4 details the body location and sensor configuration used in these studies.

**Table 1:** Summary of selected research studies investigating the use of inertial sensor technology for swimming analysis. References are presented in chronological order. Details included relate to the number of participants involved and their status (E: elite, C: competitive, R: recreational), swimming strokes examined (Fc: frontcrawl; Br: breaststroke, Bk: backstroke, Bf: butterfly); accelerometer and gyroscope sensor ranges; device size and mass; volume (where three dimensions are reported); sampling rate; filter design (LP: Low Pass, BW: Butterworth, HW: Hamming window, MA: Moving average); data storage; data transmission (RF: radio-frequency, IR: infra-red); output variables reported for different phases of swimming (F: free-swimming; S: starts; T: turns) and validation procedures. (Unrep = unreported).

Ref.	Year	Participants			Swim Strokes				Sensor Range		Size & Mass	Volume	Sample Rate	Filter Design	Data Storage	Data Trans.	Output Variables	Swim Phase			Validation Methods
		E	C	R	Fc	Br	Bk	Bf	Accel. (m·s^-2^)	Gyro. (rad·s^−1^)	Size (m × 10^-3^)	(m^3^)	(Hz)		(MB)			F	S	T	
											Mass (kg × 10^-3^)										
[15]	2000	-	2	-	•				±490.5	N/A	Unrep	Unrep	Unrep	LP BW	Unrep	Unrep	stroke phase acceleration patterns	•			Video
											62										
[16]	2002	-	5	-	•	•			±98.1	±26.2	142.8 × 23	Unrep	128	Unrep	128	Unrep	stroke phase acceleration & angular velocity patterns, effect of fatigue	•			Video
											78										
[17]	2002	-	5	-	•				±98.1	N/A	88 × 21	Unrep	128	LP BW	32	Unrep	stroke phase acceleration patterns, effect of fatigue	•			Video
											50										
[12]	2003	-	2	-		•			± 490.5	N/A	Unrep	Unrep	Unrep	LP BW (10 Hz)	Unrep	Unrep	stroke phase acceleration patterns	•			Video
											62										
[18]	2004	-	1	-	•	•	•	•	±19.62	N/A	Unrep	Unrep	150	LP HW (0.5 Hz)	Unrep	IR	stroke id, lap time, stroke count	•			Video& observation
											Unrep										
[19]	2004	6	-	-	•				Unrep	Unrep	Unrep	Unrep	250	Unrep	Unrep	Unrep	Stroke id, stroke count	•			Video & observation
											Unrep										
[20]	2004	-	5	-	•	•	•	•	±98.1	±26.2	142 × 23	Unrep	128	Unrep	128	Unrep	stroke phase acceleration patterns	•			Video
											78										
[21]	2005	-	1	-	•				±19.6	N/A	Unrep	Unrep	150	LP HW (0.5 Hz)	Unrep	IR	Lap time, stroke count, stroke rate	•			Video & manual
											Unrep										
[22]	2006	-	4	-	•				±98.1	N/A	88 × 21	Unrep	128	LP BW	Unrep	Unrep	stroke phase patterns, arm joint angles	•			Video
											50										
[23]	2007	-	-	-	•	•	•	•	N/A	N/A	Unrep	Unrep	32	LP (5 Hz)	Unrep	Unrep	lap count, lap time, stroke count, swim speed, distance	•			Unrep
											Unrep										
[24]	2007	-	-	-	-	-	-	-	Unrep	N/A	Unrep	Unrep	Unrep	Unrep	Unrep	Unrep	Hip rotation	•			Unrep
											Unrep										
[25]	2008	-	4	4	•				Unrep	N/A	Unrep	Unrep	256	LP BW (0.01 Hz)	1000 Flash	Unrep	Velocity, distance per stroke	•			Manual
											Unrep										
[26]	2008	1	-	3				•	±14.7–±58.9	N/A	Unrep	Unrep	200	LP BW (10 Hz)	128 Flash	USB	stroke count, stroke rate, temporal stroke phase analysis	•			Video
											Unrep										
[11]	2008	6	-	-	•	•	•	•	±19.6	N/A	Unrep	Unrep	150	LP HW (0.5 Hz)	Unrep	IR	stroke id, lap time, stroke count, stroke rate	•			Video & manual
											Unrep										
[27]	2008	-	2	-	•	•		•	±19.6	±2.6	52 × 34 × 12	2.12 × 10^−5^	150	LP HW (0.5 Hz)	128 Flash	RF, USB	acceleration, velocity	•			Tethered speed meter
											22										
[28]	2008	-	-	-	•	•	•	•	Unrep	N/A	Unrep	Unrep	100	LP BW (2.5 Hz)	Unrep	2.4 GHz RF	velocity, stroke rate, distance per stroke, intra stroke velocity	•			Unrep
											Unrep										
[29]	2008	-	1	-	•	•	•	•	Unrep	Unrep	Unrep	Unrep	Unrep	Unrep	Unrep	Unrep	Acceleration profile recognition	•			Video
											Unrep										
[30]	2009	-	1	-	•				Unrep	N/A	36 × 42 × 12	5.14 × 10^−5^	256	Unrep	1000 Flash	Unrep	Acceleration	•			Unrep
											34										
[31]	2009	7	-	15	•				±29.4	N/A	36 × 42 × 12	5.14 × 10^−5^	256	LP BW (0.01 Hz)	1000 Flash MMC	USB	velocity, lap time, time per stroke, stroke length, orientation	•			Video & observation
											34										
[32]	2009	-	-	-	•	•	•	•	Unrep	Unrep	Unrep	Unrep	Unrep	Unrep	Unrep	Wi-Fi, Bluetooth, ANT or RF	stroke id, average speed, pace, distance, stroke count, swim distance, lap count	•			Unrep
											Unrep										
[33]	2009	12	-	-	•				±19.6	>600	52 × 33 × 11	1.89 × 10^−5^	100	LP BW (0.5 Hz)	256	USB	kick rate, kick count	•			Video
											20.7										
[34]	2009	14	-	-	•				±19.6	>600	52 × 33 × 11	1.89 × 10^−5^	100	LP BW (0.5 Hz)	256	USB	kick rate, kick count	•			Stopwatch
											20.7										
[35]	2009	-	1	-	•				Unrep	N/A	Unrep	Unrep	128	Unrep	Unrep	2.4 GHz RF	Arm acceleration and timing profiles	•			Video
											Unrep										
[36]	2009	-	-	-	•				Unrep	Unrep	Unrep	Unrep	Unrep	Unrep	Unrep	Bluetooth, ZigBee or Wi-Fi	lap counter, lap time, stroke count, stroke length	•			Unrep
											Unrep										
[37]	2009	-	-	-	•	•	•	•	Unrep	N/A	Unrep	Unrep	Unrep	Unrep	Unrep	Unrep	lap count, stroke count	•			Unrep
											Unrep										
[38]	2010	-	-	-	•	•	•	•	Unrep	Unrep	Unrep	Unrep	30	LP (1 Hz)	Unrep	USB	stroke id, stroke count, stroke rate, stroke length, lap time, speed, force	•			Unrep
											Unrep										
[39]	2010	-	-	-	•	•	•		Unrep	Unrep	Unrep	Unrep	Unrep	Unrep	Unrep	Unrep	stroke count, lap count	•			Unrep
											Unrep										
[40]	2010	-	1	-	•	•	•	•	±29.4	±8.7	150 × 90	Unrep	50	LP BW (5 Hz)	4	RF	stroke count, stroke rate, lap count	•			Video
											Unrep										
[41]	2010	-	1	-	•				±29.4	±8.7	150 × 90	Unrep	50	LP BW (5 Hz)	4	RF	stroke count, stroke rate, lap count, start and turn phase analysis	•	•	•	Video
											Unrep										
[42]	2010	-	-	-	•				Unrep	Unrep	Unrep	Unrep	Unrep	LP	Unrep	Unrep	body orientation, speed, lap time	•			Unrep
											Unrep										
[43]	2010	-	-	1	•	•			Unrep	Unrep	Unrep	Unrep	190	Unrep	Unrep	Wireless	stroke phase acceleration and angular velocity profiles	•			Unrep
											Unrep										
[44]	2010	-	-	1	•	•	•		Unrep	N/A	Unrep	Unrep	Unrep	LP (5 Hz)	2	2.4 GHz RF	pitch and roll angles, breathing patterns	•			Unrep
											7										
[45]	2010	-	1	-	•				±29.4	±8.7	150 × 90	Unrep	50	LP BW (5 Hz)	4	RF	acceleration profile during turns			•	Video
											Unrep										
[46]	2010	3	-	-	•	•	•	•	Unrep	N/A	Unrep	Unrep	100	Unrep	Unrep	Unrep	stroke id	•			Video
											Unrep										
[47]	2010	8	-	-	•	•	•	•	Unrep	Unrep	88 × 51 × 25	1.1 × 10^−4^ Unrep	100	Unrep	Unrep	Unrep	angular velocity, temporal phase assessment, stroke rate, r index	•		•	Video & stopwatch
											93										
[48]	2010	-	53	-	•				Unrep	N/A	Unrep	Unrep	Unrep	Unrep	Unrep	Unrep	speed, swim distance	•			Manual
											Unrep										
[49]	2010	-	-	-	•	•	•	•	Unrep	Unrep	Unrep	Unrep	Unrep	Unrep	Unrep	RF	stroke id, lap time, stroke count	•			Unrep
											Unrep										
[50]	2011	-	1	-				•	±14.7–±58.9	Unrep	Unrep	Unrep	200	LP BW (0.6 Hz)	512 Flash	USB	acceleration, angular velocity, pitch angle	•			Video
											Unrep										
[51]	2011	12	-	-	•				±19.6	>600	52 × 33 × 11	1.89 × 10^−5^	100	LP BW (0.5 Hz)	256	USB	kick rate	•			Video
											20.7										
[52]	2011	-	-	1	•				Unrep	N/A	Unrep	Unrep	50	Unrep	Unrep	RF	stroke phases	•			Unrep
											Unrep										
[53]	2011	1	-	-	•				±78.5	±26.2	52 × 33 × 10	1.72 × 10^−5^	100	LP HW (0.5 Hz)	1000	2.4 GHz RF	temporal stroke phase analysis	•			Video
											20										
[54]	2011	-	-	-	•				Unrep	Unrep	Unrep	Unrep	100	Unrep	Unrep	2.4 GHz RF	Unrep	•			Unrep
											Unrep										
[55]	2011	-	-	6	•				Unrep	Unrep	Unrep	Unrep	200	Unrep	Unrep	Unrep	simulated arm stroke patterns	•			Video
											Unrep										
[56]	2011	2	-	-	•				±78.5	±26.2	52 × 33 × 10	1.72 × 10^−5^	100	LP HW (0.5 Hz)	1000	2.4 GHz RF	turn phase acceleration patterns			•	Video
											20										
[57]	2011	-	2	-	•	•	•	•	±29.4	±8.7	150 × 90	Unrep	50	LP BW (5 Hz)	4	RF	stroke count, stroke rate, stroke duration, lap count	•			Video
											Unrep										
[58]	2011	-	-	-	•	•	•		Unrep	N/A	Unrep	Unrep	50	Unrep	Unrep	Unrep	stroke id	•			Unrep
											18										
[59]	2011	-	11	-	•	•	•		Unrep	N/A	Unrep	Unrep	50	MA	Unrep	Unrep	stroke id, stroke count, swimming intensity	•			Unrep
											Unrep										
[60]	2011	-	1	-	•	•		•	Unrep	Unrep	57 × 91 × 24	1.24 × 10^−4^	50	Unrep	Unrep	2.4 GHz RF	stroke id	•			Unrep
											65.6										
[61]	2011	-	-	1	•				±78.5	±26.2	53 × 33 × 10	1.75 × 10^−5^	100	LP HW (0.5 Hz)	1000	2.4 GHz RF	mean velocity	•			Tethered speed meter
											20										
[62]	2012	7	-	11	•				±29.4	N/A	36 × 42 × 12	1.81 × 10^−5^	256	LP BW (0.01 Hz)	1000 Flash MMC	USB	velocity, lap time, time per stroke, stroke length, orientation	•			Video & observation
											34										
[63]	2012	12	-	-	•				±19.6	>600	52 × 33 × 11	1.89 × 10^−5^	100	LP BW (0.5 Hz)	256	USB	kick rate, kick count, breathing patterns	•			Video
											20.7										
[64]	2012	11	-	19	•				±107.9	±15.7	Unrep	Unrep	500	Unrep	Unrep	Unrep	instantaneous velocity, mean velocity	•			Tethered speed meter
											Unrep										
[65]	2012	-	-	-	•	•	•	•	Unrep	Unrep	Unrep	Unrep	Unrep	Unrep	Unrep	Unrep	lap count, swim distance	•			Unrep
											Unrep										
[66]	2012	-	-	-	•	•	•	•	Unrep	N/A	Unrep	Unrep	Unrep	Unrep	Unrep	Unrep	stroke rate	•			Unrep
											Unrep										
[67]	2012	-	-	-	•	•	•	•	Unrep	Unrep	Unrep	Unrep	Unrep	LP 0.5–5.0 Hz	Unrep	Unrep	stroke id	•			Unrep
											Unrep										
[68]	2012	-	1	-	•				±29.4	±8.7	150 × 90	Unrep	50	LP BW (1 Hz)	4	RF	start and turn phase acceleration patterns, stroke count, stroke duration	•	•	•	Video
											Unrep										
[69]	2012	1	-	-	•				±29.4	±8.7	150 × 90	Unrep	50	LP BW (1 Hz)	4	RF	turn phase acceleration patterns, temporal analysis			•	Video
											Unrep										
[70]	2012	9	-	-	•				±78.5	±26.2	52 × 33 × 10	1.72 × 10^−5^	100	HW FIR (0.5 Hz)	1000	2.4 GHz RF	arm symmetry, stroke rate	•			Video
											20										
[71]	2013	-	2	-	•	•	•	•	±29.4	±8.7	150 × 90	Unrep	50	LP BW (1 Hz)	4	RF	stroke count, stroke rate, lap count	•			Video
											Unrep										
[72]	2013	-	20	-	•				±107.9	±15.7	50 × 40 × 16	3.2 × 10^−5^	500	LP (100Hz)	Unrep	microSD	mean velocity	•			Tethered speed meter
											36										
[73]	2013	-	6	6	•				±107.9	±15.7	50 × 40 × 16	3.2 × 10^−5^	500	LP (100Hz)	Unrep	microSD	energy expenditure, velocity, cycle velocity variation	•			Indirect calorimetry, lactate
											36										
[74]	2013	-	7	-		•			±98.1	±15.7	50 × 40 × 16	3.2 × 10^−5^	100	Unrep	Unrep	Unrep	stroke phase acceleration patterns	•			Video
											36										
[75]	2013	-	-	1	•	•	•	•	Unrep	N/A	Unrep	Unrep	50	Unrep	2	RF	stroke rate	•			Unrep
											Unrep										
[76]	2013	-	-	1	•				Unrep	N/A	Unrep	Unrep	50	Unrep	2	2.4 GHz RF	stroke count, stroke length, stroke rate, velocity	•			Unrep
											Unrep										
[77]	2013	-	-	1	•	•	•	•	Unrep	N/A	Unrep	Unrep	50	Unrep	2	2.4 GHz RF	stroke rate	•			Unrep
											Unrep										
[78]	2013	-	12	-	•	•	•	•	±14.7	±8.7	Unrep	Unrep	200	MA	Unrep	SD	stroke id	•			Video
											Unrep										
[79]	2013	-	-	1	•				±29.4	±8.7	150 × 90	Unrep	50	LP BW (5 Hz)	4	RF	block time, entry time, kick initiation time, stroke initiation time, kick rate, stroke rate, stroke count		•		Video
											Unrep										
[80]	2013	-	-	-	•	•	•	•	Unrep	Unrep	Unrep	Unrep	200	Unrep	Unrep	Bluetooth	stroke id	•			Unrep
											Unrep										
[81]	2013	1	1	-	•				Unrep	±1500	Unrep	Unrep	100	LP BW (2 Hz)	Unrep	Unrep	body roll velocity	•			Video
											Unrep										
[82]	2013	1	2	4	•				±58.9	N/A	69 × 28 × 07	1.59 × 10^−5^	100	HW FIR (0.5 Hz)	Unrep	Unrep	push-off velocity			•	Tethered speed meter
											15										
[83]	2013	8	9	-	•				±78.5	±26.2	53 × 33 × 10	1.75 × 10^−5^	100	LP HW (0.5 Hz)	1000	2.4 GHz RF	mean velocity, stroke rate	•			Tethered speed meter
											20										
[84]	2013	-	53	-	•				Unrep	N/A	29 × 37 × 11	1.18 × 10^−5^	32	Unrep	Unrep	Unrep	speed, distance	•			Stopwatch
											34										
[85]	2014	-	-	3	•	•	•		±19.6	N/A	5 × 58 × 25	7.25 × 10^−6^	Unrep	Unrep	Unrep	Bluetooth	stroke count, kick count, symmetry	•			Unrep
											Unrep										
[86]	2014	-	21	-	•	•		•	Unrep	N/A	Unrep	Unrep	100	Unrep	Unrep	2.4 GHz RF	stroke count, mean velocity	•			Video
											Unrep										
[87]	2014		9	9	•				±107.9	±15.7	50 × 40 × 16	3.20 × 10^−5^	500	LP (100 Hz)	Unrep	microSD	energy expenditure, velocity, kick rate	•			Indirect calorimetry, lactate
											36										
[88]	2014	-	-	-	•	•	•	•	Unrep	Unrep	Unrep	Unrep	Unrep	Unrep	Unrep	Unrep	stroke count, stroke id, lap count, lap time	•			Unrep
											Unrep										
[89]	2014	-	2	-	•	•	•	•	±19.6	±4.4	16 × 12 × 10	1.92 × 10^−6^	100	MA	NOR flash memory 64	433 MHz RF	stroke id, breathing patterns	•			Unrep
											Unrep										
[90]	2014	-	-	-	•	•	•	•	Unrep	Unrep	Unrep	Unrep	Unrep	Unrep	Unrep	2.4 GHz RF	lap count	•			Unrep
											Unrep										
[91]	2014	-	-	-	•	•	•	•	Unrep	Unrep	Unrep	Unrep	Unrep	Unrep	Unrep	Unrep	swim distance, lap count, lap time, stroke id	•			Unrep
											Unrep										
[92]	2014	-	-	60	•				Unrep	N/A	Unrep	Unrep	Unrep	Unrep	Unrep	Unrep	energy expenditure	•			Cosmed
											Unrep										
[93]	2014	-	45	-	•	•	•	•	±19.6	N/A	Unrep	Unrep	32	Unrep	Unrep	Unrep	stroke id	•			Video
											Unrep										
[94]	2014	-	1	-				•	±9.8	±8.7	53 × 32 × 19	3.22 × 10^−5^	Unrep	Unrep	Unrep	Blue-tooth	joint angles during fly kick	•			Video
											Unrep										
[95]	2014	-	1	1		•			Unrep	Unrep	Unrep	Unrep	Unrep	LP Fourier (8 Hz)	Unrep	Unrep	joint angles	•			Video
											Unrep										
[96]	2014	10	-	-	•	•	•	•	Unrep	N/A	30 × 30	Unrep	100	LP (2 Hz)	Unrep	Unrep	stroke id	•			Manual
											33										
[97]	2015	-	8	7		•			±107.9	±15.7	50 × 40 × 16	3.2 × 10^−5^	500	LP (100Hz)	Unrep	microSD	mean velocity	•			Tethered speed meter
											36										
[98]	2015	-	-	3	•				Unrep	Unrep	Unrep	Unrep	50	Unrep	Unrep	Unrep	Positioning	•			Video
											Unrep										

## 4. Discussion

### 4.1. Parameters for Analysing Free-Swimming

#### 4.1.1. Stroke Phase Analysis

In 2000, Ohgi and colleagues were the first to apply inertial sensor technology to identify swimming stroke phases during frontcrawl swimming from a wrist-worn accelerometer device sampled at128 Hz [15,17]. This work was soon expanded to include an analysis of other swimming strokes and also to combine the acceleration signal with angular velocity measurements from a gyroscope [12,16,20]. During a swimming stroke, a swimmer will continuously alter shoulder, elbow and wrist joint angles, combined with actions of the rest of the body, to change hand position in the water and generate propulsive forces. This movement can be tracked by analysing the signal signatures from these inertial sensors and through comparison with video footage.

**Figure 5:**
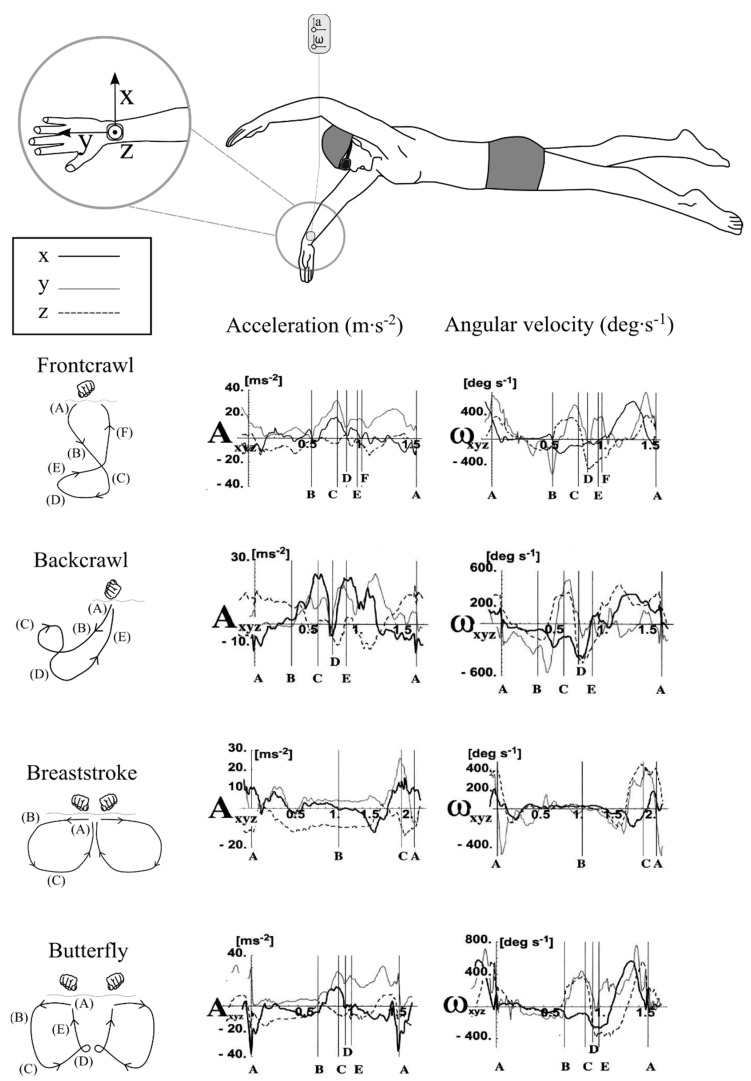
Different swimming styles will exhibit different acceleration (A) and angular velocity (ω) patterns. Representative signal output from the wrist is shown. Each signal begins from the point of hand entry into the water and the various phases of each stroke style are identified with vertical lines. Characteristic features of each signal allow researchers extract key performance related information. Adapted from Maglischo [1] and Ohgi [20].

For example, a positive local acceleration maximum in the ulnar-radial direction (X-axis) seen in Figure 5 is indicative of the start of the insweep, which is followed by local minimum along the distal-proximal direction (Y-axis) at the beginning of the upsweep phase during frontcrawl [15]. These studies found that wrist acceleration will range from -40 m∙s^−2^ to +40 m∙s^−2^ whilst angular velocity will range from −10.5 rad∙s^−1^ to +14.0 rad∙s^−1^, with evident differences between strokes (Table 2). This early research confirmed that features of the acceleration signal output could potentially be used as a novel means of analysing a swimmers technique.

**Table 2:** Indicative range of acceleration and angular velocity values recorded at the wrist during each of the four swimming strokes. Adapted from Ohgi [20].

Swimming Stroke	Acceleration (m·s^−2^)	Angular Velocity (rad·s^−1^)
Frontcrawl	−20 to +40	−7.0 to +8.7
Backstroke	−10 to +30	−10.5 to +10.5
Breaststroke	−20 to +40	−7.0 to +7.0
Butterfly	−40 to +40	−7.0 to +14.0

**Figure 6:**
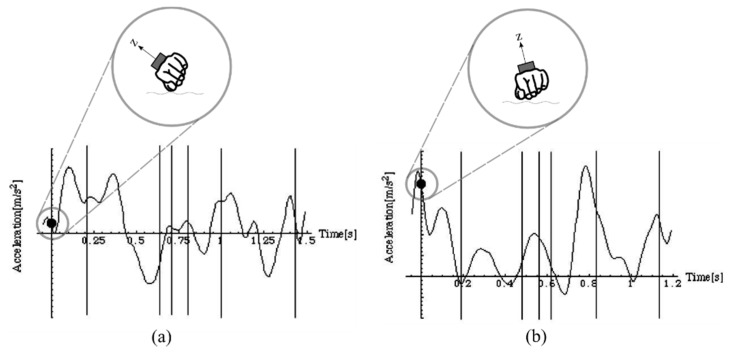
Features of the acceleration signal can be used to distinguish between different swimming techniques. Swimmer (**a**) demonstrates a more ideal pitch angle at the point of hand entry to the water and this is reflected in the Z-axis (palmar-dorsal) acceleration of approximately 0 m∙s^−2^. In contrast, swimmer (**b**) has a much larger Z-axis acceleration at this point, which is indicative of a flatter hand entry to the water. Adapted from Ohgi, Yasumura, Ichikawa and Miyaji [15].

Additionally, this work highlighted an individual nature to signal signatures, albeit with limited subject numbers. To illustrate, Figure 6 compares the Z-axis acceleration profile for two swimmers during a frontcrawl stroke cycle. This palmar-dorsal direction can be related to the orientation of the wrist. Differences in the signals can be seen throughout the different phases. For example, it can be seen in Figure 6a that the Z-axis is close to 0 m∙s^−2^ at the point of hand entry (at time zero). Conversely, the value at the same point in the stroke is much larger in Figure 6b. Ohgi, Yasumura, Ichikawa and Miyaji [15] postulated that this difference can be explained by the two swimmers displaying a different pitch of the hand at the point of entry, with swimmer (a) displaying a more ideal pitch as opposed to swimmer (b) who demonstrated a flatter hand entry. Furthermore, it has been found that the effects of fatigue can be seen in the acceleration signal. Reduced acceleration during the upsweep phase is indicative of poor elbow extension and this can be related directly to shorter stroke durations and reduced propulsive movements during the arm sweeps [17]. Differences such as these facilitate a detailed and specific analysis of a swimmers hand actions, but also lead to difficulties in identifying common features upon which to base automatic feature detection algorithms.

**Figure 7:**
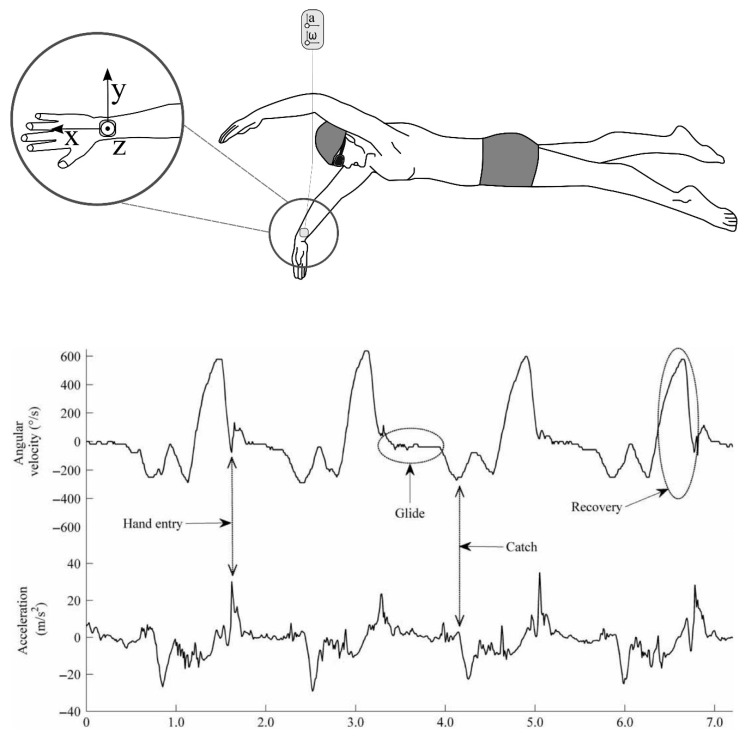
Comparison of signal output from both gyroscope and accelerometer sensors for four arm strokes. The signal displayed is from the Y-axis (ulnar-radial direction). It can be seen that the angular velocity pattern that is obtained is smoother and may facilitate easier feature detection of key events such as hand entry; glide; catch; and recovery. Reproduced with permissions from James, Leadbetter, Neeli, Burkett, Thiel and Lee [53].

An Australian research group, led by Davey and James, later combined the signals from both an accelerometer and a gyroscope in an attempt to more accurately define the phases of arm action during frontcrawl [53]. These events were identified and described through visual inspection of the sensor data in conjunction with video images. This work compared arm, back and leg worn sensors and argued that the primary signal of interest for stroke phase detection should be the medio-lateral signal of the gyroscope located on the wrist, which is indicative of pronation and supination of the forearm (Figure 7) Acceleration data were then used as a secondary confirmation of specific events such as the instant of hand entry. The authors acknowledged a previously highlighted issue that the point of hand exit from the water, marking the beginning of the recovery phase, was not easily identified and did not correspond with any particular spike in any of the three dimensional accelerometer or gyroscope sensor signals. Indeed, Ohgi and colleagues had combined the upsweep and recovery phases when determining the temporal durations of phases of arm actions for this reason [17]. This issue also raises concerns about feasibility testing of new technology using dry-land swim bench apparatus, as found in Lee, Burkett, Thiel and James [55], as the acceleration signal may not be consistent with that produced in the water, even if stroke patterns are reproducible.

A recent paper has suggested a possible solution for this. By using multiple sensors positioned on both forearms and on the swimmers lower back, researchers measured the changing angle between the sensors at the sacrum and the forearm throughout the stroke, calculated from the angular velocity signal. It was suggested that the start of the recovery phase occurs when this angle is at a maximum value of approximately 2.6 to 3.1 rad (150°–175°), and a peak detection algorithm was used to track these points in the stroke [10].

**Figure 8:**
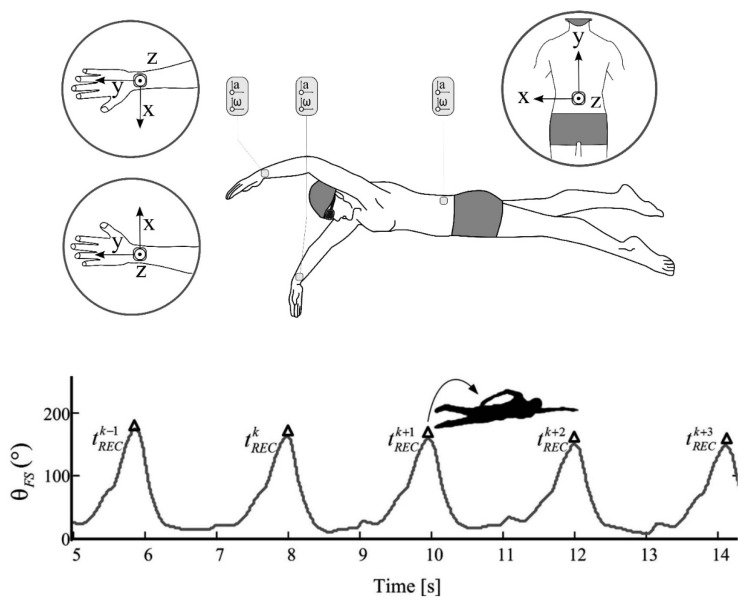
The changing angle between the Y-axis of sensors worn at the sacrum and on the forearm, measured using the gyroscopic signal and used to determine the start of the recovery phase, which occurs when the angle is at a maximum value. Reproduced with permissions from Dadashi, Crettenand, Millet, Seifert, Komar and Aminian [10].

Furthermore, the authors developed a change detection algorithm to track the changing slope from both the accelerometer and gyroscope signals and were able to identify stroke phases as a result (Figure 8). By using sensors on both arms, this work also allowed for the measurement of the lag time between propulsive phases, termed the index of coordination (IdC), which previous research has found to correspond with skill level and swimming intensity and is traditionally measured using video [4,99,100]. The results demonstrated the validity of this approach, with a strong linear relationship found between the sensor derived data and the gold-standard data determined from video footage.

The research undertaken investigating how stroke phases can be determined using inertial sensors is important because it has provided coaches with a new way of analysing swimming techniques. This work has also demonstrated the potential for examining movement characteristics of both left and right arms independently [35] or to determine stroke rates and other performance related variables from regularly occurring patterns in the sensor signal, laying the foundations for future exploration in this field.

#### 4.1.2. Stroke Type Identification

**Figure 9:**
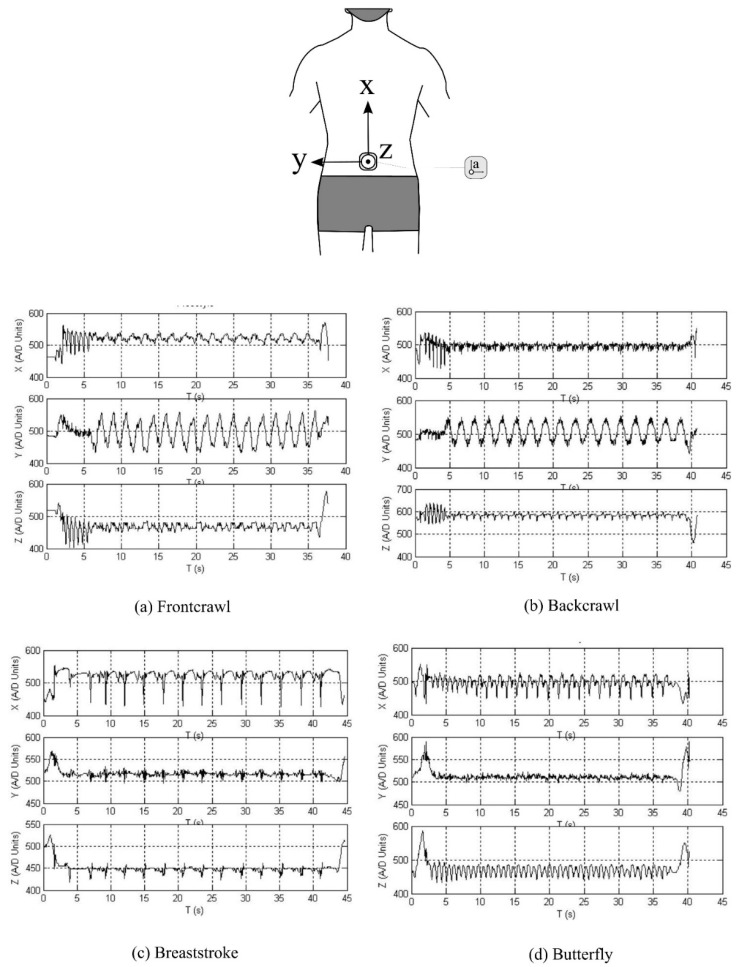
Sample acceleration output from a lower back worn sensor for each of the four competitive swimming strokes. Characteristic patterns of each stroke can be used to automatically identify stroke styles. The A/D (analog to digital) units referred to can be related to acceleration, such that 512 A/D units is representative of 0 g. Values greater than 512 A/D units are therefore positive g-values and values less than 512 A/D units are negative g-values. Reproduced with permissions from Davey, James, Anderson [18].

Specific characteristics of the acceleration profile for the four competitive swimming strokes allow for swimming stroke type to be detected. Similar methodological approaches have been described in the literature that have detected stroke type using sensors positioned on the upper or lower back [11,27,59,79,101,102], wrist [27,32,59,91], chest [85,93] and head [78,89]. Figure 9 provides a representation of a typical acceleration signal from the lower back over a full lap of swimming for each stroke [79]. A swimmer will lie in a supine position when performing backstroke. Consequently, the Z-axis signal (*i.e.*, acceleration in the anterio-posterior direction) outputs a value of approximately +1 g (+9.81 m∙s^−2^) during backstroke. This is in contrast to the other three strokes in which the Z-axis tends towards −1 g (−9.81 m∙s^−2^) as the swimmer is in a prone position when performing these strokes and the device will be orientated in the opposite direction. Additionally, whilst the X and Y axes during all four strokes appear to show similarities, there are differences in the magnitude and spread of the local maxima and minima that can be recognised.

**Figure 10:**
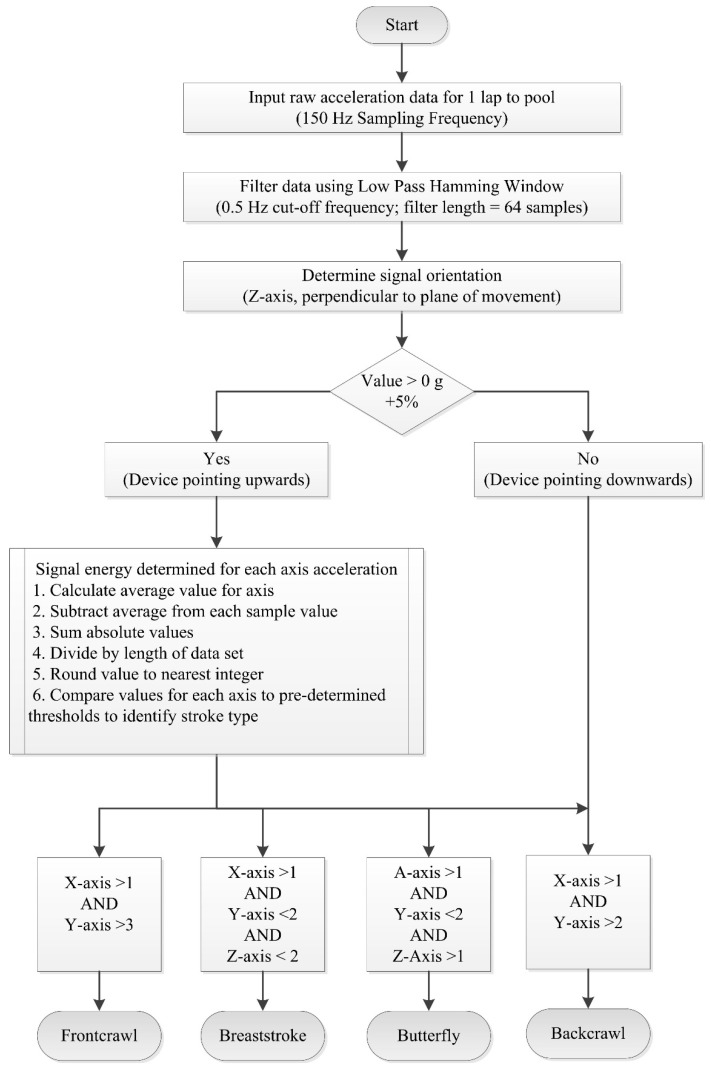
Flowchart for a stroke identification algorithm used to distinguish between each of the four competitive swimming strokes. Adapted from Davey, Anderson and James [11].

Researchers have exploited these characteristics to develop methods which may be used to automatically detect the stroke type completed for any given lap [11,46,59,60]. Davey and colleagues [11,19] developed an algorithm that calculates sensor orientation and signal energy (Figure 10). The raw acceleration data were filtered using a low-pass Hamming window filter with a cut-off frequency of 0.5 Hz. The device orientation for each lap of swimming was determined using the Z-axis data as described above to first discriminate backstroke from the other three strokes. To distinguish further between strokes, thresholds were set for the three axes based on the magnitude of the filtered signal [11]. For example, it can be seen in Figure 9 that the amplitude of the Y-axis (medio-lateral direction) is large for frontcrawl and backstroke. This is because the body will rotate along this longitudinal axis during each stroke cycle. In contrast, breaststroke and butterfly are known as short-axis strokes and do not feature this rotation. Overall recognition accuracy across all strokes of 95% was reported when the data were compared to the prescribed swimming protocol. As such, it is not certain if there were any recognition issues due to specific stroke styles. Additionally, only six swimmers were included in the study so more rigorous testing of the algorithm would be necessary to offer a thorough evaluation of its reliability. That said this research did demonstrate for the first time that stroke type could be determined from the acceleration signal using straightforward signal processing and computational methods.

Siirtola, Laurinen, Roning and Kinnunen [59] utilised linear and quadratic classification methods and achieved comparable results to Davey, Anderson and James [11]. The specific details of the methodology employed went unreported but it involved a sliding window technique to process the data using a window size of two seconds with an interval of half a second between windows. What is noteworthy about the study by Siirtola, Laurinen, Roning and Kinnunen [59] is that comparisons were made of the accuracy of stroke identification: (i) for different sampling rates; (ii) between wrist and upper back worn accelerometer devices; and (iii) for three of the four competitive swimming strokes. The data were then resampled at 5, 10 and 25 Hz, to assess what effect this may have on detection accuracy. The results are summarized in Table 3 and indicate that the back worn sensor achieved better overall accuracy (95.3% at 25 Hz compared to 89.8% for the wrist). This was true at each of the sampling frequencies tested and for all three swimming styles included in the study. It is well established that the pattern of hand movement during swimming shows considerable variances owing to various factors including individual anthropometric and technique differences, skill level, swimming speed and fatigue [38,100,103]. It is possible that these variations are affecting the results of the wrist location. It was also found that sampling rates as low as 5 Hz can be used to accurately distinguish between styles and similar recognition rates were reported for each of the three strokes tested [59].

**Table 3:** Results of automatic stroke style identification, comparing different sensor locations and sampling frequencies. The back worn device produced more accurate results for all styles and sampling frequencies. Note that the results provided for the three swimming styles relate to data calculated at 5 Hz. Adapted from Siirtola, Laurinen, Roning and Kinnunen [59].

Comparison Measure	Recognition Accuracy	
	Wrist	Upper Back
Sampling Frequency		
5 Hz	88.5%	95.1%
10 Hz	88.9%	95.4%
25 Hz	89.8%	95.3%
Swimming style		
Frontcrawl	90.8%	96.1%
Backstroke	88.8%	97.1%
Breaststroke	92.6%	96.7%

**Figure 11:**
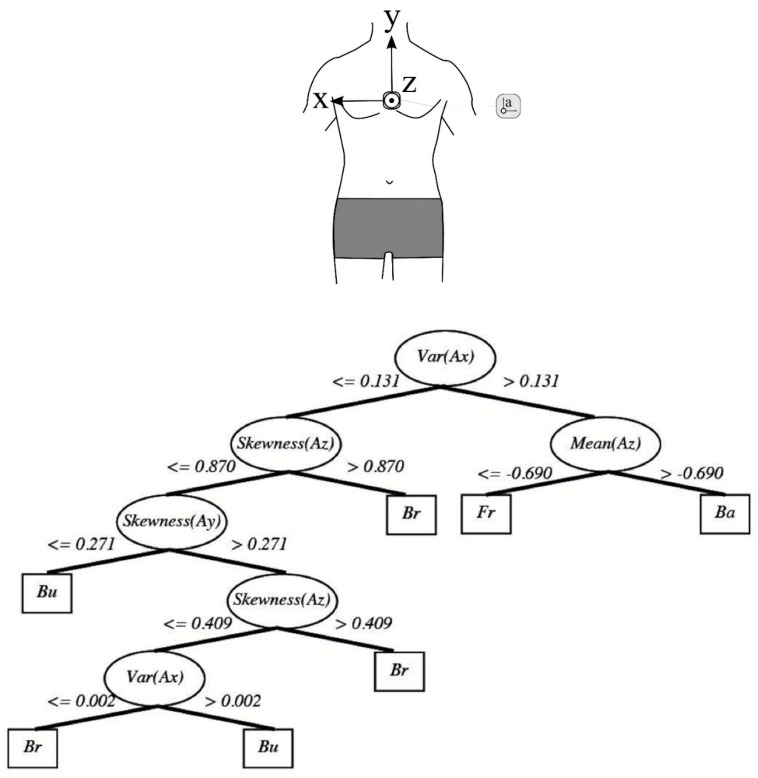
Stroke identification classification model based on descriptive statistical features of all three axes of the acceleration signal from a chest worn device. Thresholds were set to the data from each of the three axes (values in m∙s^-2^) in order to classify stroke styles. Reproduced with permissions from Ohgi, Kaneda and Takakur [93].

A recently published conference paper also using classification methods for automatic stoke identification was based on data mining procedures (neural network and decision tree) [93]. Using a chest mounted tri-axial accelerometer, descriptive information including the mean; variance and skewness of the acceleration data for all axes were examined to establish thresholds and used to distinguish between strokes (Figure 11). Results indicated a high overall accuracy (91.1%) and this approach does warrant further examination as a much larger data set was involved than in previous studies discussed. It appears that the torso offers a more accurate location for stroke style identification compared with the wrist, but this may come at a trade-off in terms of usability and user comfort. However additional investigation is warranted due to the limited research currently available. Other body locations, such as the head for example, may offer an alternative solution and convenient location.

Much of the patent literature also features automatic stroke identification functionality and this is certainly an acknowledgement of the importance of this for applied use of inertial sensors in swimming settings [32,38,49,67,91]. Unfortunately, the accuracy of the approaches in the patent literature is untested and there is often insufficient information related to the specific system specifications and signal processing techniques. For example, Yuen [49] describes a method of distinguishing strokes that replicates that of Davey, using the polarity of the Z-axis channel to distinguish backstroke and then comparing the same individual axes to further distinguish between the other styles. However, the specifics regarding the threshold values employed are not described and no data is presented to explore the accuracy of the approach. Furthermore, in most instances, several embodiments may be suggested within a given patent, providing several potential methodologies.

**Figure 12:**
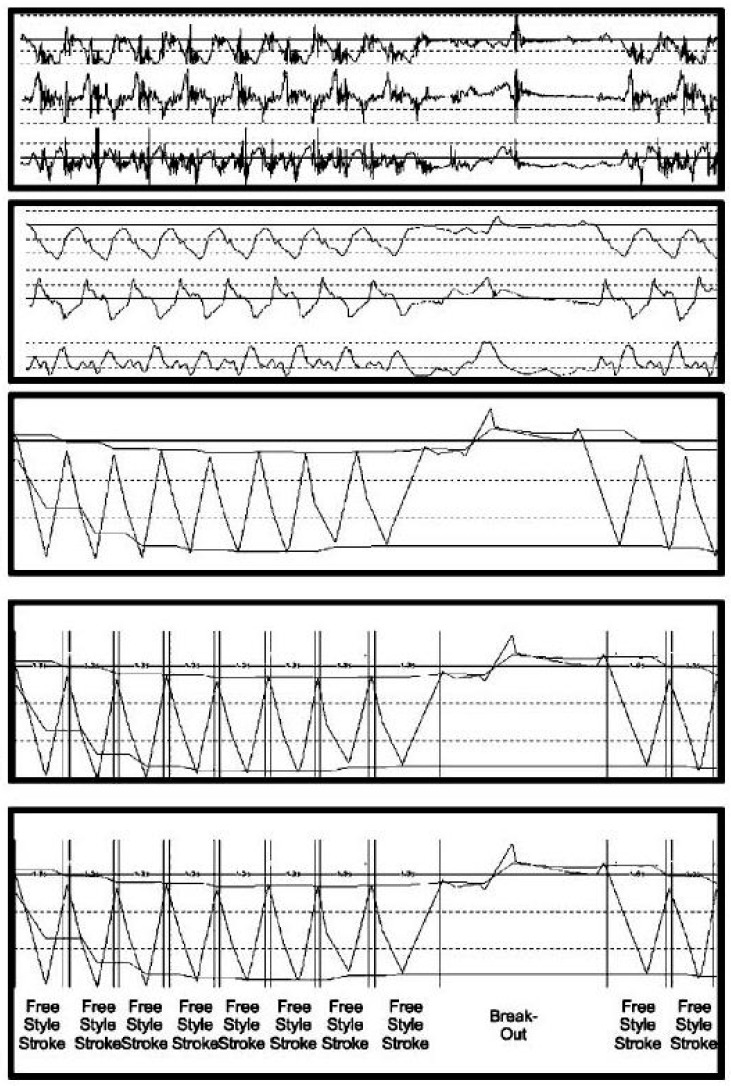
The process of determining stroke type from a wrist worn tri-axial accelerometer device: (**a**) raw acceleration signal; (**b**) low-pass filter with a cut-off of 1 Hz; (**c**) peak detection algorithm used to isolate maxima and minima; (**d**) individual strokes are identified; (**e**) recognition models applied to determine stoke type. Adapted from Anthony and Chalfant [38].

One such example of this ambiguity is provided in Figure 12 [38], which describes the process of determining stroke type from a wrist worn tri-axial accelerometer device. In part (a), the raw acceleration signal is recordedat 30 Hz. A low-pass filter is applied with a cut-off of 1 Hz (b). In part (c) a peak detection algorithm is used to isolate maxima and minima along the x-axis, representing acceleration in the direction of swimming. This is achieved using a moving window technique with a window size of 1.5 s. Individual strokes are identified in part (d), using heuristic techniques, such as determining a sequence of maxima-minima-maxima. It is suggested that a threshold of greater than 1 g (9.81 m∙s^−2^) in total acceleration within a three second duration is used, but it is not clear if these same sequences and values may be applied to all stroke types. Finally, in part (e), recognition models are applied to determine which of the competitive swimming types is involved. However, various possible options for conducting this process are mentioned, including linear discriminants, hidden Markov models and neural networks, but with no data presented to test any of these approaches.

Where reported, automatic stroke type identification algorithms appear to show good levels of accuracy and can be readily incorporated into embedded systems for applied use. However, this feature is not included in most research designs. This could be because the majority of studies are concentrated solely on frontcrawl and as such, no detection algorithm is necessary. Even where multiple strokes are included, study protocols are prescribed in advance so the sensor output can be manually attributed to a specific stroke [57,76,86,101]. Whilst this may be expected of early exploratory research work in this area, it does call into question the robustness of these devices for use in applied settings, where all four strokes are used interchangeably, even for elite swimmers with specific stroke specializations. The requirement for the end user to manually input the swimming stroke completed for a given lap or training interval severely hampers the functionality of these systems. Additionally, without clear details of the methodology employed, it is difficult for researchers to fully assess the merits of any given approach or to arrive at a best-practice methodology for identifying stroke type.

#### 4.1.3. Lap Time

**Figure 13:**
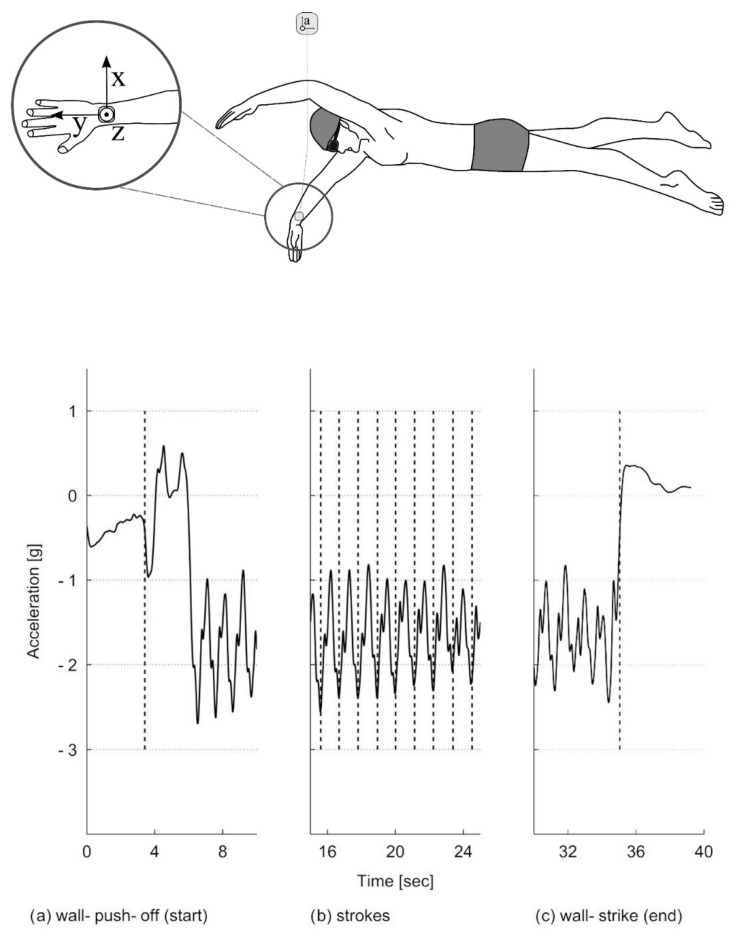
Lap times can be determined by identifying events at the pool walls. Both push-off and wall strike events result in rapidly changing slopes and a corresponding signal amplitude that exceeds that observed during mid-pool swimming. Reproduced with permissions from Bächlin and Tröster [62].

The ability to record lap times during swimming allows for the intensity of effort to be monitored closely. Measuring lap time requires the detection of events when the swimmer makes contact with the pool walls. Bächlin and Tröster [62] filtered the acceleration signal from the longitudinal axis of a wrist worn device using a low-pass 2nd order Butterworth filter with a cut-off frequency of 0.01 Hz. The resultant filtered data was used to determine events at the pool walls (Figure 13). A push-off was registered at the point of the first falling slope in acceleration, whereas a large impact peak and rising slope signified that a wall strike had occurred. The authors reported that values were within ±0.3 s of the criterion measure. Unfortunately, significance was not reported and the criterion used was a manual method using a stopwatch which itself is prone to human error.

**Figure 14:**
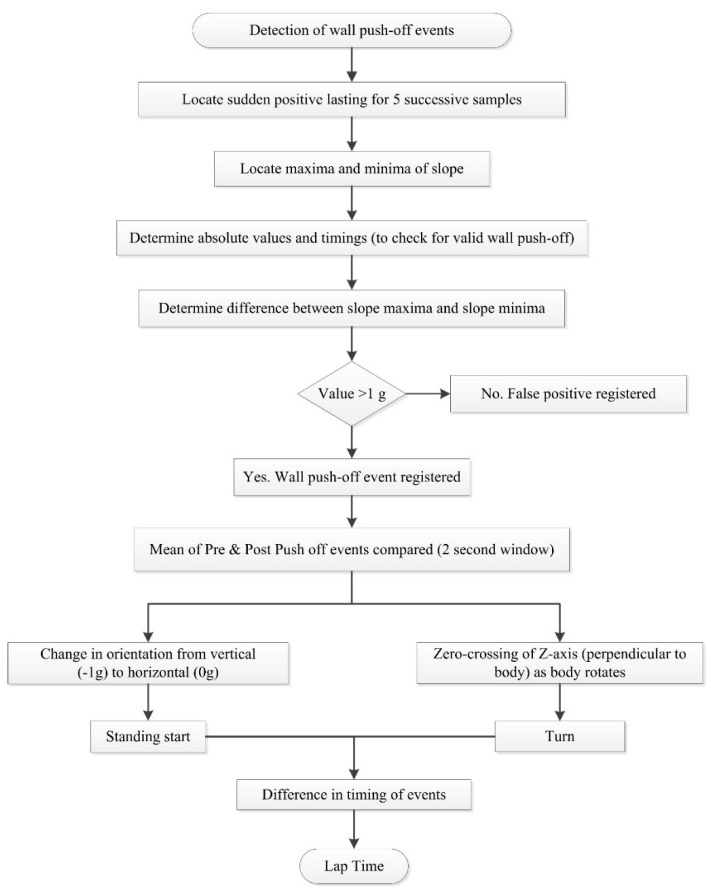
Flowchart for a lap time detection algorithm based on detection of wall push-off events. Adapted from Davey, Anderson and James [11].

Davey, Anderson and James [11] describe algorithms for detecting two distinct types of wall push-off events with an accelerometer worn on the lower back, those following the commencement of swimming and those after turns (Figure 14). As the swimmer commences swimming from a standing start, a change in orientation from vertical to horizontal can be recognised. A turn can be detected using a zero-crossing algorithm about the perpendicular axis as the swimmer rotates in the water [11,57]. Additionally, the wall push off is characterised by a rapid increase in acceleration over a short interval, such as a 1 g rise (9.81 m∙s^−2^) over a 0.1s duration. However, Davey, Anderson and James [11] reported a significant difference (p < 0.01) existed in lap time calculations between the video and that of the accelerometer device (mean difference −0.32 ± 0.58 s). Further analysis revealed that this was as a result of errors in the part of the algorithm that was used for the detection of the commencement of swimming as opposed to the algorithm used to detect turns or the end of the final lap. Lap time differences for the first 100 m of a 200 m swimming trial averaged −0.38 ± 0.23 s (significantly different at p < 0.01) whilst no significant differences reported for the second 100 m of the trial (0.05 ± 0.45 s). The authors also reported that the offset was consistent, with the accelerometer tending to underestimate lap times [11].

An additional concern with the detection of wall contact is that the arms or legs will absorb the majority of the impact [11,59], causing difficulty in setting threshold values for automatic detection of turns and the end of a swimming interval, especially with a sensor positioned on the torso. Furthermore, some have reported issues with detecting peaks during turns owing to individual differences in turning technique, such as gliding into the wall on approach [59]. Impact accelerations will be more clearly visible from wrist worn sensors at the end of a swimming interval [62] and during butterfly and breaststroke turns but the opposite is true during frontcrawl and backstroke as the arm will not make wall contact when performing flip turns.

It appears that the accurate determination of lap times using inertial sensors remains an area of ongoing research. Further empirical testing is necessary to ensure accuracy of this important parameter. The ability to detect wall contact events, and thus record lap times, is paramount, not just from a coaching point of view but also as many other variables are derived from this parameter such as average speed, stroke count, stroke rate and stroke length.

#### 4.1.4. Swim Distance

**Figure 15:**
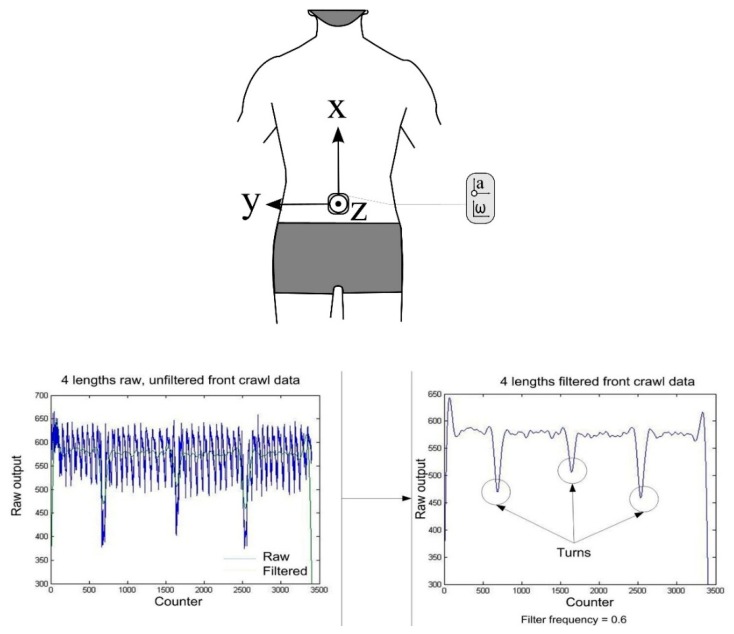
Turns performed during frontcrawl can be automatically detected by thresholding of the filtered acceleration signal from the axis perpendicular to the plane of movement as this undergoes a rapid change in acceleration as the swimmer rotates during the tumble. Reproduced with permissions from Le Sage, Bindel, Conway, Justham, Slawson and West [40].

The same methodology described above for identifying events at the pool walls to measure lap times can also be used in a more simple fashion to register that a lap has occurred. Subsequently, by knowing the length of the pool, swim distance is readily calculated by utilizing a lap counter function that is not dependant on determining the exact instant of wall contact or push-off. For example, Le Sage, Bindel, Conway, Justham, Slawson and West [57] describe a lap counter algorithm that tracks when turns have been registered. Figure 15 shows how this was achieved. The raw acceleration data from the Z-axis (perpendicular to the plane of movement) was filtered using a low-pass Butterworth filter with a cut-off frequency of 2 Hz for frontcrawl swimming. The filtered data show clear local minima which are indicative of the swimmers transverse rotation during the flip-turn. A simple threshold was applied to these data to facilitate automatic counting of the laps performed [40,57]. This process appears to be quite robust due to the clear amplitude difference observed during the turn but data were only provided for four consecutive laps of swimming so this requires further verification. Others did report an 88.9% accuracy in detecting that a turn had occurred using a similar process and using a slightly larger data set comprising of 12 swimmers each completing 400 m of swimming in total [78].

Interestingly, Wright and Stager [84] recently reported an alternative method of recording swimming distance that does not rely on determining when events at the pool walls have occurred or prior knowledge of the pool length. Using a regression technique, the authors reported a statistically significant relationship between raw accelerometer output and actual swim distance completed (R^2^ = 0.9608, p < 0.05), using a combination of wrist and ankle worn devices. This promising technique requires further study as the effects of different swimming styles are unknown but one drawback is that it cannot be used to determine lap times.

Swim distance is also probably of little importance in an elite swimming environment whereby training distances are prescribed by the coach in advance as part of the training plan. However it may have a useful application in open water swimming as an alternative to GPS tracking. Additionally, swimming distance is a more important functional consideration for sensor based systems designed for recreational swimmers, who do not have the benefit of a coach to monitor their training. In fact, this function may be used by some users as the primary determinant of whether training goals had been achieved, in much the same way as a recreational runner will wish to know the distance completed during a run without necessarily wanting to know any other information about the activity. Hence there is a greater prevalence of lap counter and swim distance functions in the patent literature [30,32,36,39,60,65,90,91,104].

#### 4.1.5. Stroke Count and Stroke Rate

The most commonly calculated variables from inertial sensor devices are stroke count and stroke rate [11,19,26,28,32,38,39,57,59,62,65,71,77,83,85,86], both key performance indicators in competitive swimming [1]. The back and wrist are the most prevalent locations and Table 4 shows that a similar approach to stroke count measurement can be taken at both body sites and this approach typically involves the detection and summation of acceleration peaks for a given lap.

**Figure 16:**
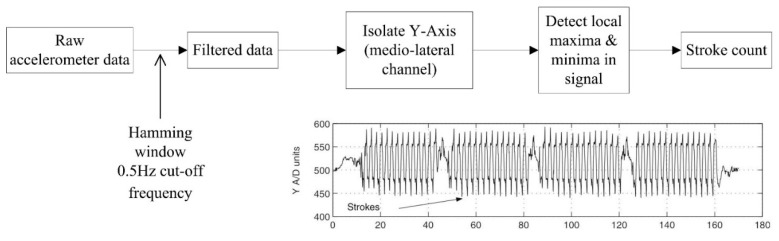
The regularly repeating pattern of swimming exhibited allows for a stroke count algorithm based on tracking peaks and troughs in the acceleration signal. Reproduced with permissions from Davey, Anderson and James [11].

Davey, Anderson and James [11] isolated the medio-lateral acceleration signal (Y-axis) of a back worn device and identified peaks and troughs in the signal (Figure 16). This characteristic waveform is representative of the roll of the body about that axis and as such the strokes completed can be determined. The authors programmed their device to find the first peak and not count another peak until a trough had been registered. The results show very high recognition rates for stroke counts within one stroke of the criterion data [11,71]. This suggests that the body roll action used may not always be indicative of an arm action, especially at the beginning and end of laps. Anthony and Chalfant [38] argue that similar issues may also arise from a single wrist worn device as the sensor will have to make an assumption regarding the movement of the other arm.

**Table 4:** Details of various methods used for the detection of stroke count using inertial sensor devices, with validation methods and reported detection accuracy.

Ref.	Stroke Count Detection Method	Sensor Location	Protocol	Accuracy
[11]	Peak detection of medio-lateral acceleration signal	Lower back	N = 6; 4 × 50 m intervals (164 data sets analysed) Video and manual data used for comparison	All strokes: 90% ± 1 of actual. Frontcrawl: 65% accuracy, 100% ± 1 of actual.
[26]	Peak detection of anterio-posterior acceleration signal and zero-crossing of longitudinal signal	Lower back	N = 4; 4 × 25 m intervals of butterfly Video used as criterion measure	97.6% accuracy
[59]	Peak detection of acceleration signal with different threshold levels for each stroke. Different axes used for different strokes	Wrist & upper back	N = 11; Intervals completed at various speeds (up to 1053 data sets); Validation method not reported	All strokes: >99% accuracy
[62]	Peak detection of forward acceleration signal	Wrist	N = 18; 7 × 50 m frontcrawl intervals; Video and manual data used for comparison	Not reported
[71]	Zero crossing of acceleration signal with thresholding. Medio-lateral axis for frontcrawl and backstroke. Forward axis for breaststroke and butterfly	Lower back	N = 2; 4 × 25 m each stroke	All strokes: 56% accuracy, 100% ± 1 of actual.
[86]	Peak detection of acceleration signal; GPS integration necessary	Head	N = 21; 3 × 100 m swims (1 each of butterfly, breaststroke & frontcrawl); Video data used for comparison	Butterfly: r = 1.00 (p < 0.05); Breaststroke: r = 0.99 (p < 0.05); Frontcrawl: stroke count was “not discernible” due to sensor location

**Figure 17:**
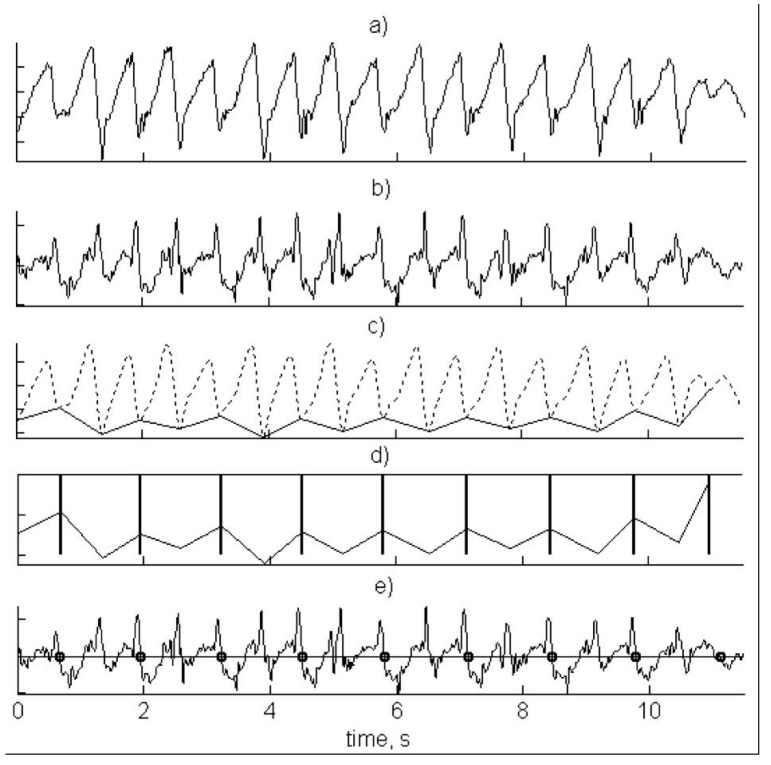
Stroke count detection method using back worn accelerometer: (**a**) raw vertical axis acceleration; (**b**) raw anterio-posterior axis acceleration; (**c**) filtered vertical axis acceleration; (**d**) filtered vertical axis acceleration with envelope applied; (**e**) stroke detection on anterio-posterior axis using peaks in envelope. Reproduced with permissions from Daukantas, Marozas and Lukosevicius [26].

Subsequently, some studies chose to use multiple acceleration channels in an attempt to improve recognition accuracy [26,59,71]. Figure 17 describes the steps in this process used in one example for butterfly swimming [26]. The anterio-posterior axis signal is filtered using a 4th order low-pass Butterworth filter with a 10 Hz cut-off frequency. Local minima of this filtered signal are determined to create an envelope. The maxima of this envelope are then used to approximate the location of each stroke on the longitudinal axis and a zero-crossing algorithm of this axis is completed to identify the exact instant when each stroke begins. The authors reported an accuracy of 97.6% for strokes recorded by four swimmers each performing 100 m butterfly swimming.

Recent attempts to determine stroke count using a using a head mounted device have also been made [86]. Again a peak detection method was used to automatically count strokes completed, although the actual axis used for analysis was unspecified. Excellent accuracy was reported for butterfly and breaststroke swimming (Table 4). However, during frontcrawl and backstroke, swimmers will aim to keep their heads as static as possible and consequently the signal output did not demonstrate the patterns of repeated peaks and troughs to facilitate accurate stroke count recognition. Additionally, swimmers will use different breathing patterns which may not be synchronous with arm actions, further complicating this approach. The study was exploratory in nature and further investigation of a head worn device is warranted, including a thorough analysis of all three acceleration axes, to attempt stroke counting for all four swimming strokes. The inclusion of a gyroscopic signal may also aid this investigation. A head-mounted position has clear advantages for ease of positioning and is found to be quite unobtrusive to the swimmer in comparison to other locations.

**Figure 18:**
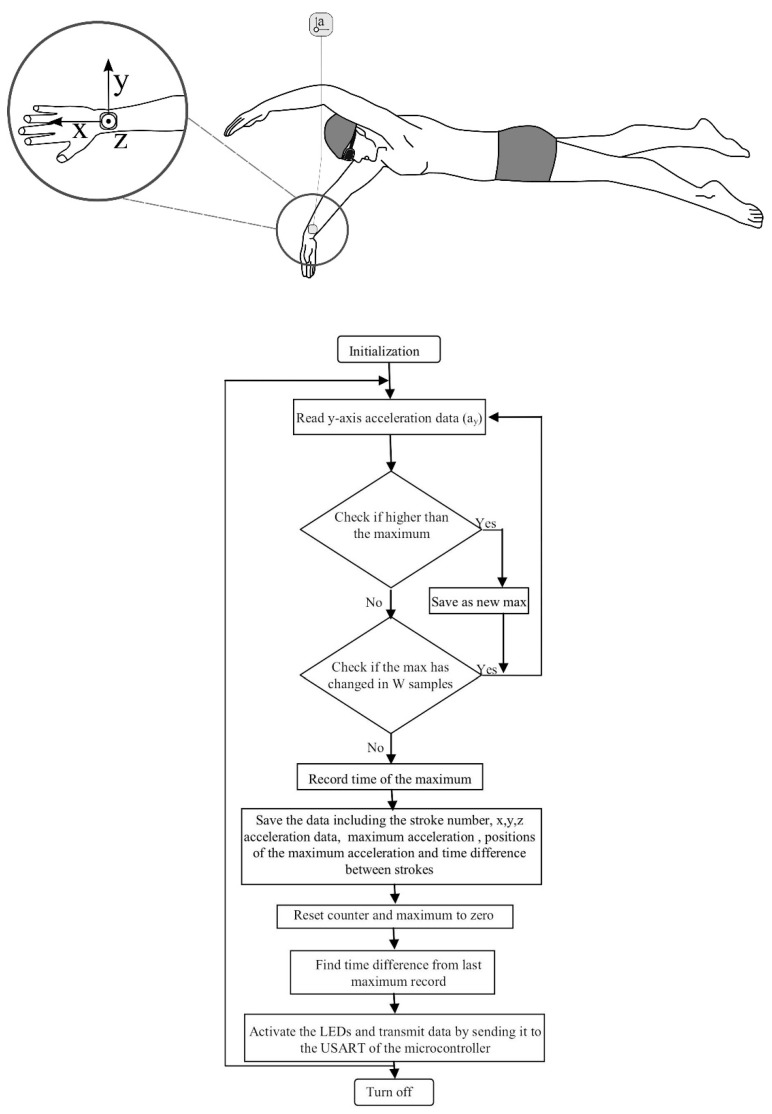
Flowchart of a stroke rate detection and transmission algorithm used to provide real-time feedback to a swimmer. Stroke rate is detected using a wrist worn accelerometer and information is provided to the swimmer via an LED based receiver system located in the goggles. Reproduced with permissions from Hagem, O’Keefe, Fickenscher and Thiel [77].

One study evaluated the accuracy of a zero-crossing algorithm for measuring stroke rate by comparing the performance of the algorithm against manually digitized video footage [71]. Differences with the criterion measure ranged from −0.25 strokes per minute (breaststroke) to +0.19 strokes per minute (backstroke). Within-subject reliability testing also showed positive results, although with low subject numbers. The interclass correlation coefficient for butterfly ranged from +0.74 to 0.91, with standard error of the mean of 1.2 to 1.6%. Finally, stroke rates over four lengths of frontcrawl were compared. The overall average was the same for both automatic and manually derived data (33.5 strokes per minute) although small differences were observed when each length was compared in isolation. Hagem, O’Keefe, Fickenscher and Thiel [77] suggested that this approach is overly complex and requires additional processing in comparison to peak detection methods. Figure 18 provides an overview of their alternative methodology which involved the transmission of stroke rate values from a wrist worn accelerometer device to a receiver in the swimmers goggles to facilitate real-time feedback on performance [77]. However a thorough evaluation of the accuracy of this algorithm is not reported. Earlier work had investigated the accuracy of a peak detection based stroke rate measurement algorithm, comparing with both manually counted and video derived data, albeit for frontcrawl only [11]. Results showed a magnitude and spread of error similar to reference values. The stroke rate algorithm was accurate to within one stroke of the manually collected data for 90% of data sets.

At present, these algorithms all appear to determine stroke rate over the full lap of swimming, whereas the common convention in applied practice would be to calculate this parameter over three stroke cycles performed mid-pool to better reflect actual stroke rate during free-swimming [1]. An algorithm could be derived to facilitate a similar approach to bring these methodologies in line with coaching practices.

#### 4.1.6. Swimming Velocity

Swimming velocity is a key performance indicator that has recently become the focus of attention in several studies [62,64,83,86,97], with a range of methodologies for its calculation previously reported (Table 5). In one study, mean velocity was calculated using the time taken to swim a known pool length of 50 m [62]. The authors compared this automatic parameter extraction method against a standard manually calculated protocol involving repeated 50 m frontcrawl intervals with increasing velocity [105], with analogous results. However, manually calculated velocity was found to be lower than the automatic method. A possible explanation for this lies in the effects of increased velocity following the wall-push off when measured over the full 50 m pool length. An alternative approach negates this by only measuring velocity over a shorter mid-pool distance, thus the influence of the wall push off is excluded. Hagem, Thiel, O’Keefe and Fickenscher [76] calculated velocity by dividing stroke length by stroke rate. In this instance, the velocity measurement is more reflective of the speed achieved during the free-swimming phase.

**Table 5:** Details of various methods used for the detection of swimming velocity using inertial sensor devices and reported detection accuracy.

Ref.	Swimming Velocity Detection Method	Sensor Location	Accuracy
[62]	Average speed determined as time taken to cover known pool distance, recorded with accelerometer.	Wrist	1.67% upper bound error in velocity calculations
			1.33% upper bound error in stroke duration calculations
[64]	Trapezoidal integration of forward acceleration. Geometric moving average change detection algorithm to account for integration drift. Determined both instantaneous and average velocity.	Lower back	Instantaneous velocity: RMS error = 11.3 cm·s^−1^
			Average velocity: Spearman’s Rho 0.94 (p < 0.001)
[72]	Gaussian process framework	Lower back	RMS error = 9.0 cm·s^−1^, r = 0.95 (p < 0.001)
[83]	Integration of acceleration signal with correction based on swimmers height. Five points on different axes and resultant acceleration determined	Lower back	1.08 m·s^−1^: bias 0.01 m·s^−1^; limits of agreement: −0.26 to 0.29 m·s^−1^ (94.75% of data points inside limits of agreement) 1.01 m·s^−1^: bias 0.02 m·s^−1^; limits of agreement: −0.17 to 0.20 m·s^−1^ (96.25% of data points inside limits of agreement)
[84]	Regression analysis and predictive equations based on output of two accelerometers	Wrist & ankle	r = 0.76, R^2^ = 0.57, SEE = 0.14 m·s^−1^ (p < 0.001)
[86]	GPS positioning. 5 point moving average to smooth. Exclusion criterion included for manual inspection of velocity data.	Head	Butterfly: SEM = 0.18, 95% CI = 0.14–0.27 (Sig. difference with criterion, p < 0.05)
			Frontcrawl: SEM = 0.13, 95% CI = 0.10–0.19 (No sig. difference)
			Breaststroke: SEM = 0.12, 95% CI = 0.09–0.17 (No sig. difference)
[97]	Bayesian linear regression (BLR) compared against Linear least square estimator (LLS) and Gaussian process regression (GPR)	Lower back	LLS: RMS error = 17.7%, 14.4 cm·s^−1^, r = 0.56 (p < 0.001)
			GPR: RMS error = 9.2%, 6.1 cm·s^−1^, r = 0.91 (p < 0.001)
			BLR: RMS error = 9.7%, 6.2 cm·s^−1^, r = 0.91 (p < 0.001)

Another recently described method for calculating swimming velocity involves integration of the acceleration signal. Studies have attempted to validate this approach using back worn sensors and a tethered speed-meter as reference [27,64,83]. In one study, mean velocity was determined using peak detection algorithms for specific channels to identify five key data points in the acceleration signal [83]. Results of a Bland-Altman analysis indicated that mean velocity recordings were within 4% of the reference values and integration error was determined to be non-significant (0.002 m·s^−1^). Nonetheless, others have questioned the repeatability of this approach due to issues associated with resolving the sensors orientation with respect to gravity [97].

**Figure 19:**
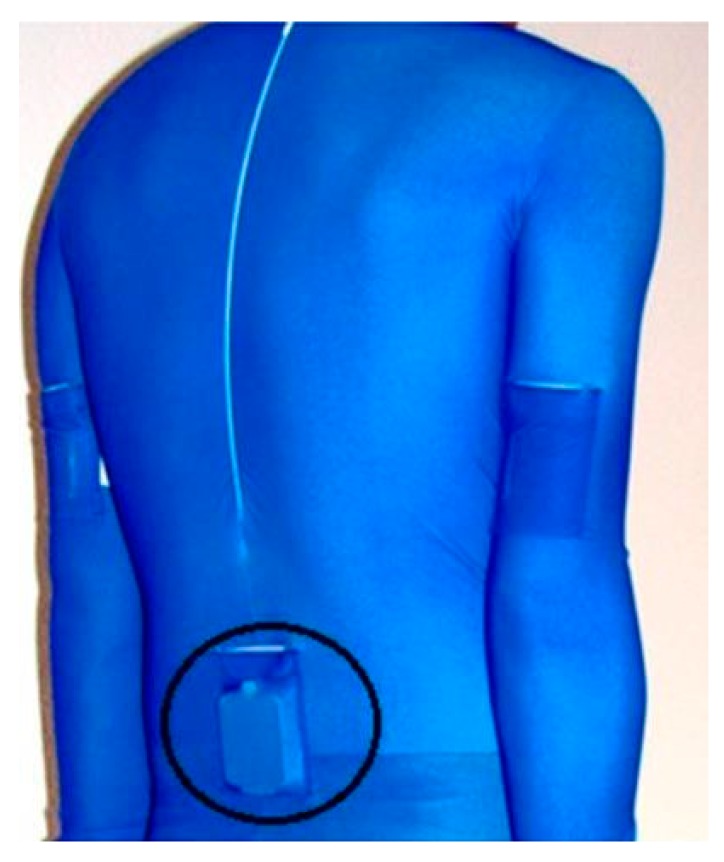
A modified swim suit design allows for accurate positioning of the sensor device but may result in unwanted sensor movement. Reproduced with permissions from Dadashi, Millet and Aminian [97].

Instantaneous and mean velocity has also been determined using a geometric moving average change detection algorithm to account for integration drift. A two-fold validation procedure was completed and similar mean velocity accuracy to Stamm, James and Thiel [83] was reported (3.5%). Instantaneous velocity displayed an RMS difference of 0.113 m·s^−1^, a relative error of 9.7% compared to the reference value [64]. The authors noted that some of the error may have been attributed to movement artefact owing to the modified swim suit design employed (Figure 19). Interestingly, the determination of instantaneous velocity allowed for intra-cycle velocity variations (IVV) to be assessed and the authors demonstrated that this variation is visible on the acceleration trace and can distinguish between elite and non-elite swimmers.

Recently, the same authors extended their investigations and compared different mathematical regression models for the determination of swimming velocity as an alternative to integration [72,97]. Results for both Gaussian and Bayesian regression methods are comparable with a relative error of 9.2% and 9.7% respectively. In contrast to earlier methods, these models do not require prior knowledge of the pool length, extending their applicability in real-world settings. Additionally, Bayesian regression can be performed without requirements for the inclusion of constraints related to the swimming stroke performed [97]. For example, the Gaussian method was tested during frontcrawl swimming and the algorithm assumes that the sacrum will roll about the longitudinal axis in a uniform manner so modifications would be necessary for other swimming strokes [72].

Much of this work has to date used a tethered speedometer as the criterion measure so results are only verified over a single lap of swimming at present [64,72,83,97]. Tethered systems may also interfere with kicking action, further complicating the procedure. Two additional approaches have been reported for velocity measurement which overcome this constraint, but both have other disadvantages [84,86]. A recent study describes using accelerometry as a means of quantifying training load in competitive swimmers [84]. The algorithm involved the summation of raw accelerometer output from both wrist and ankle worn sensors, which were found to correlate positively with swim velocity and distance. Predictive equations were validated following linear regression analysis and showed a significant correlation between actual and predicted values for both distance and velocity, indicating that this approach may offer a sound method of quantifying velocity in applied settings. However, the authors note that there is a necessity for specific regression equations to be customised for individual swimmers, which would be essential for accurate measurements, requiring future experimental investigation.

#### 4.1.7. Kick Count and Kick Rate

Quantifying a swimmers kicking pattern is a relevant concern for coaches as the action of the lower limbs will help maintain body positioning, aid streamlining and contribute to propulsion [106]. Moreover, kicking patterns can be difficult to observe, even with underwater video, as the movements are rapid and water turbulence can obscure a coach’s view. One author argued that kicking patterns may be observed on the medio-lateral axis of a back worn accelerometer [85]. However, no evidence was presented and it is unclear how the distinction would be made between the actions of the arms and legs in this instance. A more plausible approach to investigating leg action is to position the inertial sensor directly to the lower limb.

**Figure 20:**
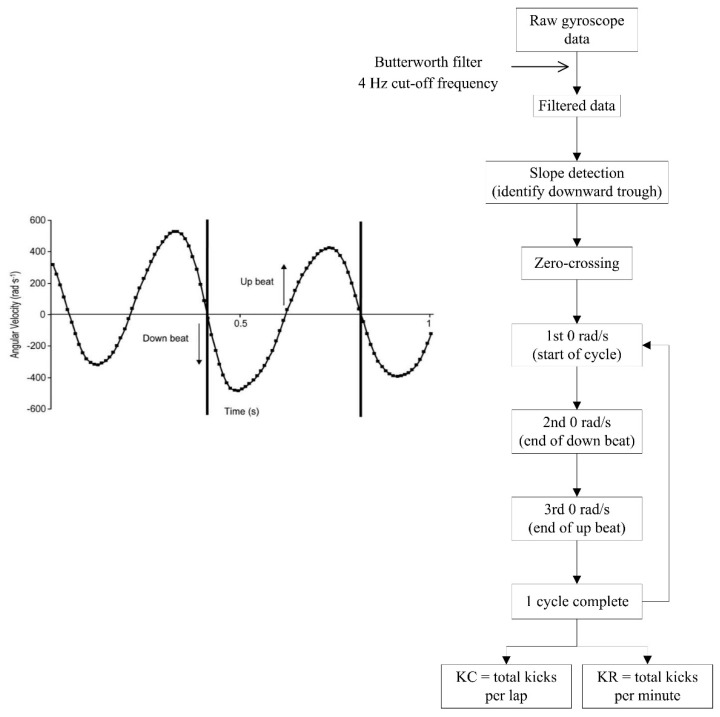
Process flowchart for detecting kick count and kick rate from angular velocity signals. Reproduced with permissions from Fulton, Pyne and Burkett [33].

Fulton, Pyne and Burkett [33] utilised a gyroscope for this purpose, as opposed to analysing the acceleration signal, and assessed the reliability and validity of the process. Angular velocity of the lower limb was found to fluctuate in the range of approximately ±600 rad·s^−1^ during the upbeat and downbeat phases of the frontcrawl kicking action and a zero-crossing algorithm was used to detect each kick (Figure 20). The results indicated that the kick count measurements during frontcrawl swimming were correlated positively with the criterion values (r = 0.96, 90% confidence interval 0.95 to 0.97) and that the standard error of the estimate (SEE) for kick count, expressed as a coefficient of variation, was 5.9% ± 0.5%.

However, a single inertial sensor placed on the anterior or lateral sides of the swimmers’ lower limb was found to be both uncomfortable and to interfere with streamlining [33]. A posterior placement on the leg however did not inhibit kicking movements and also allowed for clearer signal transmission. Researchers therefore positioned sensors on the calf of the dominant kicking leg in subsequent studies, but the effect of location on the subjects’ comfort went unreported [51].

Fulton, Pyne and Burkett [34] next quantified kick count and kick rate in Paralympic swimmers and found that decreases of almost 11% in kick rate owning to fatigue were associated with diminished overall swimming times. Meanwhile, another study by the same research group aimed to optimise kicking patterns and found that a kick rate of approximately 150 kicks per minute were associated with peak swimming speed in a similar cohort of swimmers [51]. This study additionally evaluated the inclusion of inertial sensor technology as part of a combined, integrated performance monitoring system for use in elite swimming, which has being described elsewhere recently by others also [53,71]. Notwithstanding the fact that kicking patterns were only investigated for frontcrawl swimming, it is likely that a similar algorithm could be used to accurately examine kicking in other strokes.

#### 4.1.8. Joint Angular Kinematics

**Figure 21:**
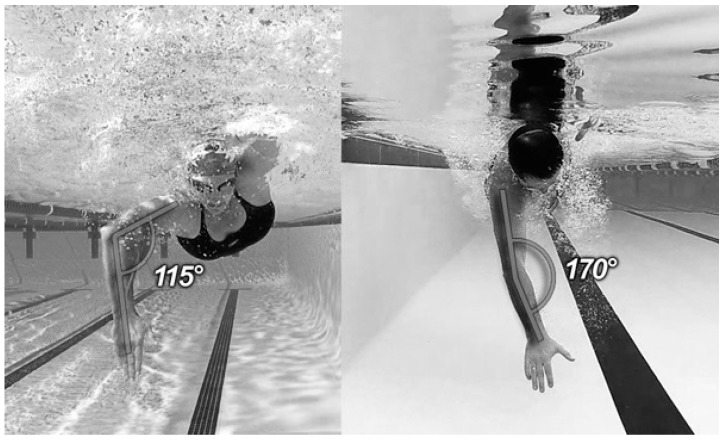
Comparison of different elbow angles produced during the insweep phase of frontcrawl swimming. Measuring these angles allows coaches to optimise technique and maximise propulsive force generation [109].

The ability to measure joint angles during swimming is important to ensure that the correct movement patterns are performed; to monitor streamlining and to maximise propulsive forces [107,108]. Important angle measurements include the elbow, shoulder and knee joints, as well as the pitch and roll angles of the torso. For example, Figure 21 compares the elbow angle of two swimmers during the in-sweep phase of frontcrawl. Previous research has shown that this elbow angle is important for maximising force production. It is suggested that elbow flexion of about 105° is optimal during this phase [107]. Therefore, whilst both of the swimmers in Figure 21 have an elbow angle greater than 105°, reducing the effectiveness of their stroke, the swimmer on the left has an elbow flexion much closer to what a coach would consider ideal. It can be difficult for a coach to observe these movements appropriately as they occur underwater and are fast moving so methods for obtaining this data are likely to be of significant interest to the coaching community.

**Figure 22:**
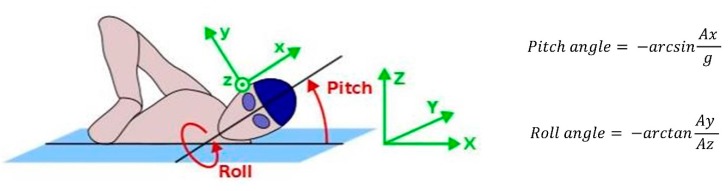
Determination of pitch and roll angles using a head mounted sensor. Reproduced with permissions from Pansiot, Lo and Yang [44].

A limited number of examples of using inertial sensor technology to measure joint angles can be found in the literature [22,44,50,94,95]. Single sensor units have been used to determine the pitch and roll angles of the swimmer using positions on the head [44] and back [50] (Figure 22). These may be calculated from the measured acceleration signal using trigonometric functions as shown. The pitch angle is important as it relates to the swimmers streamlining in the water. Additionally, the roll angle has been used to examine the effects of different breathing patterns [44]. Interestingly, Daukantas, Marozas, Lukosevicius, Jegelevicius and Kybartas [50] used complementary filters in their algorithm to determine pitch angle. The acceleration signal was low-pass filtered, whilst the gyroscopic data were high-pass filtered. Validation methods suggest that errors in pitch angle estimation were less than 2° at a cut-off frequency of 0.6 Hz.

**Figure 23:**
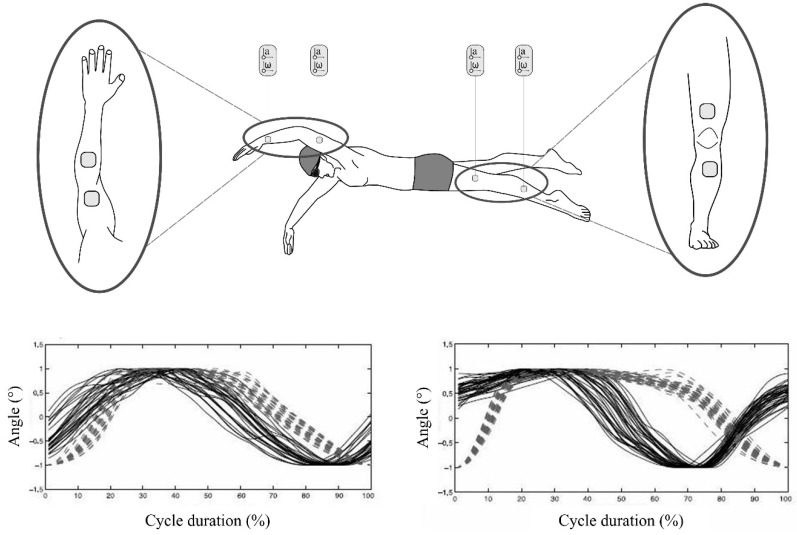
Comparison of changing joint angles produced during breaststroke stroke cycles measured using a multi-sensor system. The ideal pattern at the start of each cycle is for the knee joint (dashed line) to be at maximum flexion when the elbow joint (solid line) is near maximum extension. This is demonstrated on the right hand graph with data from an elite performer. The graph of the left hand side would be characteristic of a beginner who demonstrates near simultaneous knee and elbow movement patterns. In this example, joint angles have been normalised between −1 (maximum flexion) and +1 (maximum extension). Reproduced with permissions from Seifert, L’Hermette, Komar, Orth, Mell, Merriaux, Grenet, Caritu, Hérault, Dovgalecs and Davids [95].

Other studies have used multiple sensors to measure joint angles [94,95]. Processes typically involve methods to represent the three dimensional orientations and rotations of the swimmers limbs, including a rotation matrix [44]; Euler angles [94] or quaternions [95] and these methods have been used to analyse human movement in other sporting and health related contexts [110,111,112,113,114]. Seifert, L’Hermette, Komar, Orth, Mell, Merriaux, Grenet, Caritu, Hérault, Dovgalecs and Davids [95] demonstrated how this approach could be used to enhance the coaching process by assessing different patterns of limb coordination. Using four inertial sensors, the authors extracted knee and elbow angles during breaststroke swimming (Figure 23). The data were sampled at 100 Hz and filtered using a low pass Fourier filter with an 8 Hz cut-off frequency. Unfortunately the specific axes orientations of the sensors used was not reported. It can be seen that the less proficient swimmer (on the left in Figure 23) displays almost simultaneous knee and elbow flexion and extension, whereas a more competent performer (on the right) has near maximum extension of the elbow when the knees are at full extension, allowing for swimming speed to be better maintained throughout the stroke cycle. Seifert, L’Hermette, Komar, Orth, Mell, Merriaux, Grenet, Caritu, Hérault, Dovgalecs and Davids [95] reported a variation of between 0.09 rad and 0.15 rad from the criterion measure using this method. Phillips, Forrester, Hudson and Turnock [94] also used four sensor locations to measure joint angles, focusing on butterfly kicking technique. Using a similar method to Seifert, the results showed a very high accuracy for the knee joint (0.0019 rad accuracy) but less so for the hip joint (0.071 rad). It has been suggested that an error of 0.034 rad or less can be deemed acceptable but that errors of between 0.034 rad and 0.087 rad may require consideration when interpreting results [115].

Interestingly, the movement of the shoulder joint during swimming has not been investigated in the reviewed literature. This is surprising given the importance of shoulder kinematics for optimum stroke technique. A previous study did investigate the action of the shoulder using two inertial sensors to study the tennis serve [111]. Sensors were positioned on the upper arm and chest and comprised of a tri-axial accelerometer with a range of ±2 g (±19.62 m∙s^−2^) and uni-axial gyroscope (±5.2 rad∙s^−1^ range). Angular velocity was measured about the vertical axis and used to record shoulder abduction. A similar process could readily be applied in a swimming context although it is likely that a tri-axial gyroscope would be most appropriate in order to fully analyse all possible shoulder movements.

It is worth noting that the studies described that have measured angles are all from conference proceeding, where the level of detail is limited. Therefore, this avenue of research remains underdeveloped and it would not be advisable to draw conclusions regarding the merits or demerits of these approaches based on the limited information available. Certainly, the use of inertial sensors for measuring joint angular kinematics is commonplace in other sporting situations and high levels of accuracy have been achieved [111,113,116,117,118].

#### 4.1.9. Kinetic Variables

_ENREF_70Acceleration and deceleration signals are due to the forces exerted by the swimmer as well as the swimmer’s interaction with the environment. However, none of the reported studies in this review use accelerometers for any kinetic analysis. This is unusual, given that acceleration directly relates to force production and kinematic swimming data can be used for kinetic analysis [119]. Additionally, previous work in related fields has shown that acceleration correlates positively with peak impact force (r = 0.85, p < 0.05); average resultant force (r = 0.82, p < 0.05); and peak loading rate (r = 0.63, p < 0.05) in adults for either hip or wrist worn accelerometers [120]. Others have found a similar association, with peak ground reaction force calculated from accelerometer counts during walking and running in children [121]. This relationship has also been acknowledged in other sporting situations [117,118,122,123,124]. Meamarbashi and Hossaini [118] measured kinetic parameters such as force, torque and angular impulse with an inertial sensor system to study kicking techniques in soccer and to compare dominant and non-dominant legs, drawing clear parallels with symmetry assessment in swimming. However, force plates and pressure sensors remain the most commonly used tool for kinetic analysis in pool swimming, even for systems that employ inertial sensors [71].

It is likely that the kinetic analysis potential of sensor based systems will become more prevalent in future swimming research. Anthony and Chalfant [38] proposed that a “force-score” may be determined, for example to represent the force produced by the arm during the propulsive phase of the stroke. The process involves first determining the total acceleration (*a_total_*) from each axis of a tri-axial accelerometer (Equation 1).

(1) $$a_{total}=\sqrt{x^{2}+y^{2}+z^{2}}$$

Next, Newton’s second law of motion is used to determine the force produced, F, (Equation (2)), where *m* is the mass of the swimmers arm and *F_d_* is the drag force experienced as the arm is pushed through the water.

(2) $$F=\left(m\cdot a_{total}\right)+F_{d}$$

*Fd* is derived from the drag equation (Equation (3)), where *ρ* is the mass density of the fluid; *v* is the velocity; *C_D_* is the drag coefficient and *A* is the surface area of the arm.

(3) $$F_{d}=\frac{1}{2}\rho \cdot v^{2}\cdot C_{D}\cdot A$$

Whilst this approach appears theoretically sound, it has not been empirically tested in a swimming context and it remains unclear if such an approach would prove accurate. One area of concern is how an automatic feature detection algorithm could account for the changing anthropometric characteristics of individual swimmers. That said, should future research work validate this method of kinetic analysis, it would offer an exciting alternative to existing practices. Current methods of measuring propulsive forces generated by the action of the arms, such as 3D video analysis or the MAD system (Measurement of Active Drag) [119] require complex and expensive equipment that is not accessible to the majority of coaches.

### 4.2. Parameters for Analysing Starts

As the technology of inertial sensors continues to develop, more detailed analysis of other aspects of swimming performance, such as starts and turns, should be possible but are currently quite limited. Findings of video-based studies with elite swimmers [125,126,127] suggest that the most statistically significant starting performance variables, based on correlation with overall start time, are block time; flight time; peak horizontal velocity at take-off and peak horizontal force, and it is recommended that swimmers and coaches focus on improving these variables during training to improve overall starting performance [128]. These key variables have been measured by only one group [41,68,129].

**Figure 24:**
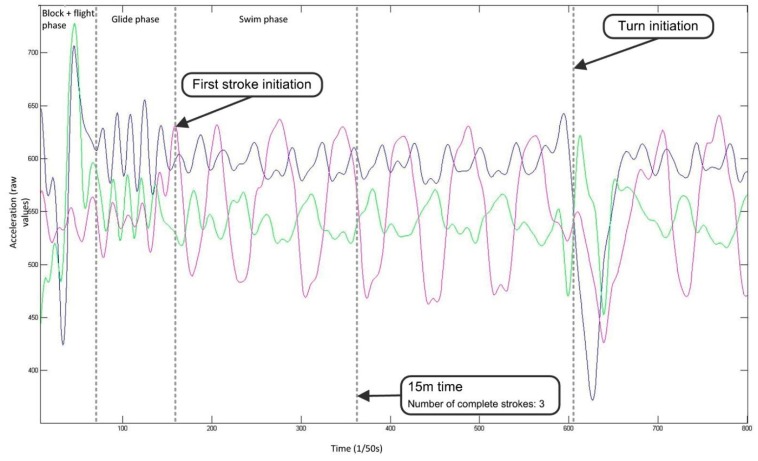
The acceleration signals from a back worn sensor device can be used to identify different phases (block, flight, glide, swim) of starts. Additional video input is necessary to determine the end of the start phase at 15 m. Reproduced with permissions from Le Sage, Bindel, Conway, Justham, Slawson, Webster and West [68].

For example in Figure 24 different phases of the start such as block, flight and glide phases were identified from the raw acceleration signal but this was only possible when the data were synchronised with video images [68], allowing for key performance related information to be extracted. Automatic detection of positional information, such as the determination of when the starting phase is completed (defined as the 15 m mark), is postulated by the authors through double integration of the acceleration signal using a Kalman filter and prior knowledge of the pool length but no empirical data has yet been published to verify this method. Another potential solution that requires further investigation is to include a photoelectric sensor to determine positional information and to help account for integration drift error [42]. Additionally, it is not clear how the phases of the start could be distinguished from these back-work sensor signals if treated in isolation. For example, there appears to be no obvious features in any of the three axes of acceleration to determine the point of entry at the end of the flight phase, based on the evidence presented thus far.

### 4.3. Parameters for Analysing Turns

**Figure 25:**
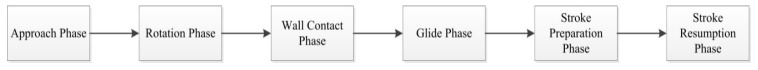
Swimming turns can be broken down into phases to facilitate a detailed quantitative analysis.

In addition to starts, turns are also a vital aspect of competitive swimming performance and have been shown to be significantly related to overall performance [16]. As a consequence, much research using video-based systems has investigated the various turning techniques [16,130,131] and coaches will spend a considerable amount of time working on turns during training. Turns are usually assessed within specific set distances, such as from 5 m before the wall to 10 m after the wall. When analysing a swimmers performance during a turn, it is also typical to break the turn down into specific phases to facilitate detailed assessment of a swimmers strengths and weaknesses and also to allow different turning techniques to be compared (Figure 25).

**Figure 26:**
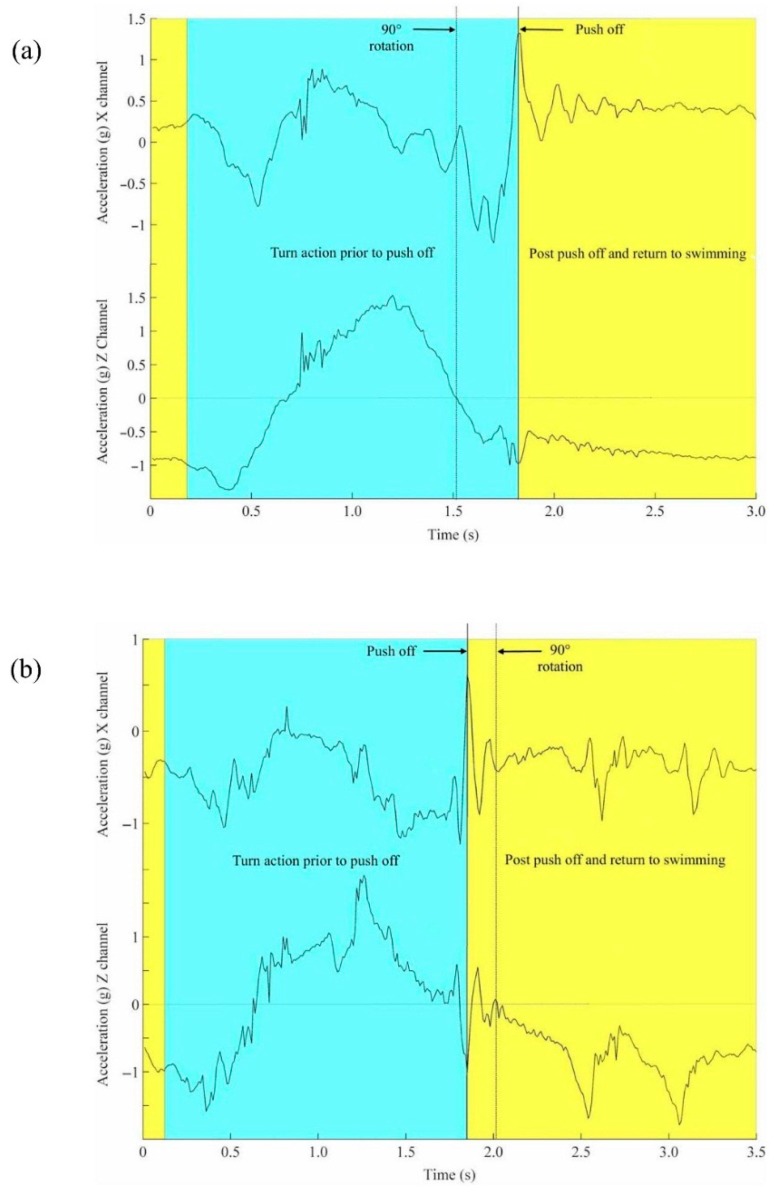
The analysis of swimming tumble turns is possible through examination of the acceleration signal. In this example, two swimmers rotation following the wall push off are compared. In (**a**), it can be seen that the swimmer has rotated by 1.57 rad (90°) before the wall push-off whilst in (**b**) the push-off occurs before the swimmer reaches 1.57 rad (90°) of rotation. Reproduced with permissions from Lee, Leadbetter, Ohgi, Theil, Burkett and James [56].

Limited studies have used inertial sensors to study turns in swimming, all using sensors positioned on the lower back [47,56,68,69,82]. One study demonstrated that key features of the frontcrawl flip turn such as the instant of wall push-off and rotation can be detected using an accelerometer [56]. It is suggested in the coaching literature that longitudinal rotation should occur after the wall push-off, in order to avoid reductions in angular velocity [1]. The researchers found that these features can be detected from an tri-axial acceleration signal sampled at 100 Hz, using the same system develop by Davey and colleagues [11] and compared the performances of two swimmers with marked differences in technique by way of example (Figure 26) [56]. The sensor was orientated such that the X-axis channel was representative of the direction that the swimmer was travelling in and was deemed to be most appropriate for recording the wall push-off. Additionally, the Z-axis (anterior-posterior direction) was chosen for analysing the rotation of the swimmer during the turn. This was a proof of concept approach to analysing turns so no further assessment was conducted, such as breaking the turn down into phases or examining if the parameters could be detected automatically using software.

**Figure 27:**
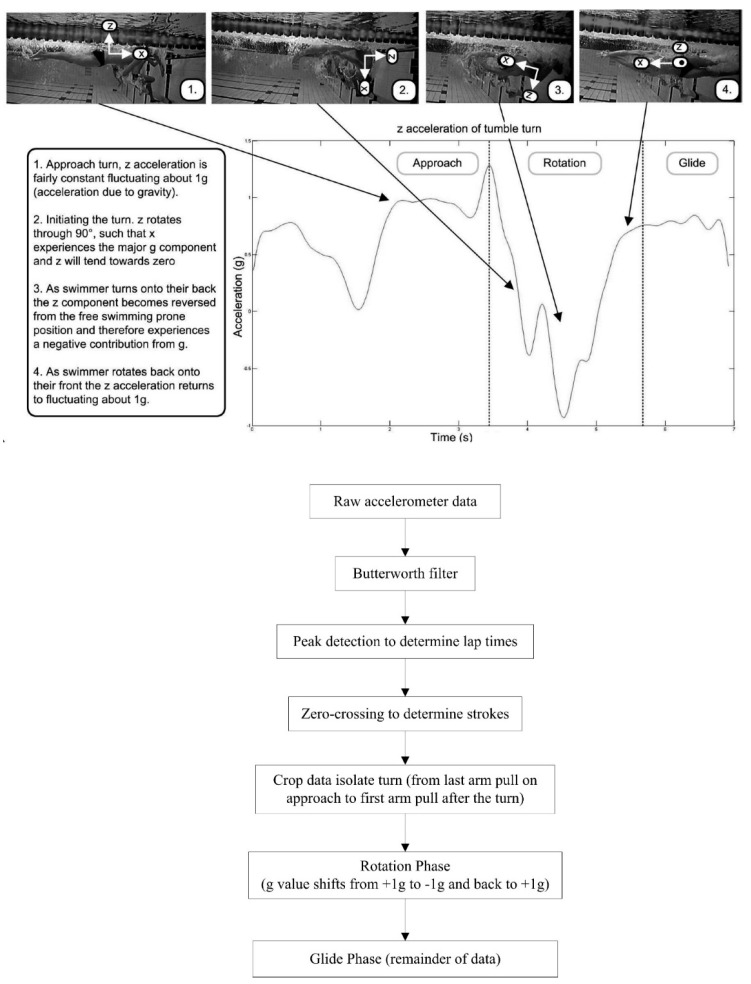
Flowchart of the process used to distinguish the approach, rotation and glide phases of the frontcrawl turn. Reproduced with permissions from Slawson, Justham, Conway, Le-Sage and West [69].

Researchers at Loughborough University went on to describe a method by which these different phases of the frontcrawl turn can be extracted from accelerometry signals [41,45,68,69,132]. The accelerometer was positioned and orientated in a similar manner to Lee, Leadbetter, Ohgi, Theil, Burkett and James [56] (Figure 27). By using both peak detection and zero crossing methods, it was possible to automatically isolate the turn during each lap by marking the point when arm movements stop and resume again. This algorithm advanced the examination of turns using sensor based systems as a temporal analysis of the different phases of a turn was now possible, albeit without the corresponding distance measurements. Variables such as time to rotation, wall contact time, glide time and stroke initiation time were measured with a high degree of accuracy, with an average difference from criterion measures of under 0.15 s [132]. Lacking from these works however is an examination of the features for other turn styles for the remaining swimming strokes, and with large groups of swimmers, as well as a lack of feature extraction methodologies to determine relevant parameters such as speed or distance.

Vannozzi, Donati, Gatta and Cappozzo [47] took an alternative approach and utilized the angular velocity signal from a tri-axial gyroscope to identify the rotation, glide and stroke resumption phases for turns performed during all four strokes. The algorithm was based on peak detection methods of analysing the signal from each of the three axes of rotation. The authors demonstrated that different signal features are indicative of different turns and also provided indicative angular velocity values for each stroke (Table 6).

**Table 6:** Angular velocity during turns. Sample data adapted from Vannozzi, Donati, Gatta and Cappozzo [47], providing indicative values of peak angular velocity (Pω) during turns performed for each of the four competitive swimming strokes.

Angular Velocity (rad·s^−1^)	Frontcrawl	Backstroke	Breaststroke	Butterfly
Pω_x_	−4.21	−6.14	−3.58	−4.01
Pω_y_	9.86	6.00	−6.61	−5.60
Pω_z_	−1.94	−0.31	−5.76	−4.54

Unfortunately, the authors did not provide any verification of their approach and there was insufficient detail regarding the signal processing methods involved. That said the study does highlight some challenges that need to be overcome before automatic feature detection of turning performance may be possible. The signal output appears to be specific to individual turning techniques. For example, the sign of the angular velocity peak (Pω) in the X and Z axes will depend on the direction of rotation. If a swimmer is performing backstroke and leads the rotation with their right arm, then Pω_x_ will be negative. However, Pω_x_ will be positive if the swimmer leads with the left arm. As seen in data in Table 6 above, Pω_x_ for backstroke for males was −6.14 rad·s^−1^. The corresponding value was +6.18 rad·s^−1^ for females in the study. This is not due to any gender differences but solely because the male participants happened to turn in one way and the females in the other direction. Furthermore, the representative peak values provided are also individually specific and will depend on other factors such as approach speed and as such no consistent pattern was discernible. This raises further challenges to setting threshold values for automatic detection. The study also highlights the importance of the Y-axis rotation in the analysis and identification of variables related to the turn as it shows a consistent pattern and will always be positive for the flip turn (performed during frontcrawl and backstroke) and negative for open turn (performed during breaststroke and butterfly). Moreover, the corresponding Pω_x_ will occur prior to Pω_y_ in backstroke and ideally after Pω_y_ in frontcrawl, further aiding automatic detection and temporal analysis.

**Figure 28:**
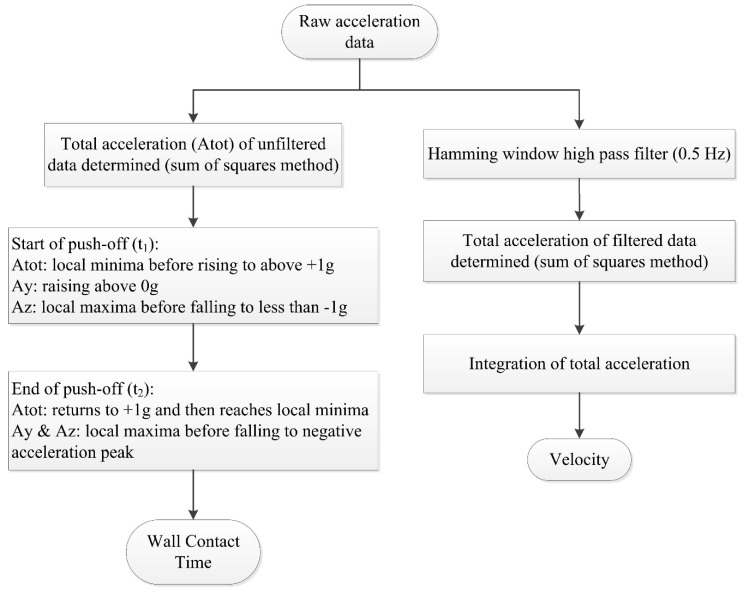
Method of determination of push-off velocity and wall contact time that utilizes all three acceleration signals and the resultant total acceleration. The raw unfiltered signal output is used to automatically determine the start and end of wall contact whilst the filtered signal was used to determine velocity during the push off phase. Adapted from Stamm, James, Burkett, Hagem and Thiel [82].

Stamm, James, Burkett, Hagem and Thiel [82] offered a novel methodology to provide a more specific analysis of aspects of the turn, using an acceleration signal to detect push-off velocity. In this study, the sensor was orientated such that the Y-axis represented the direction of travel and the total acceleration was also determined as part of the velocity determination process, which involved integration of the acceleration data (Figure 28). The researchers did highlight the potential for error using this integration method however, including issues with accumulated errors and gravitational concerns due to the changing sensor orientation, but the results provided correlated well with the gold-standard measurements. This investigation could be extended to examine how the velocity fluctuates during other phases of the turn, such as on approach and also how the velocity can be maintained through rapid butterfly leg kicks following the glide phase.

Due to the central importance of starts and turns to overall performance it is expected that this research will become more prominent in the coming years and will focus on feature extraction methods for key performance related variables. For example, a recent video-based biomechanical study provided an extensive investigation of the most statistically significant variables related to the performance of turns during frontcrawl swimming [133]. Analysing a total of 51 temporal, kinematic and kinetic variables for correlation with total turning time, the authors found that the three most statistically significant variables were: (i) maximizing the distance between the swimmers head and wall at the start of transverse rotation; (ii) a slower horizontal velocity at peak force production; and (iii) minimizing the turn distance, or 3D length of the path covered during the turn. These conclusions have been backed up by other researchers also [125,134]. The collective of studies in these sections on starts and turns have thus far been largely exploratory in nature but do demonstrate that much of this important information may possibly be extracted using sensor based systems. It is likely also that the combination of signals from accelerometers and gyroscopes represents the most sensible way forward, as has been found for the determination of free-swimming parameters.

### 4.4. Commercially Available Swimming Sensor Devices

**Figure 29:**
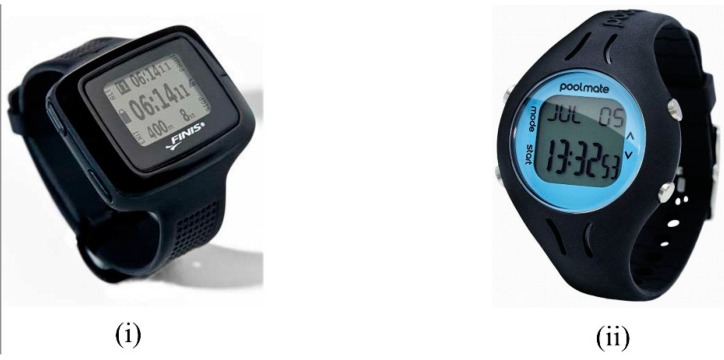
Commercially available swimming sensor devices: (**i**) FINIS *SwimSense* [136]; (**ii**) Swimovate *PoolMatePro* [138].

A number of commercially available swimming performance monitors have recently become available (examples include *AvidaMetrics*, AvidaSports LLC, Harper Woods, MI.; FINIS *SwimSense*, FINIS USA, Livermore, CA.; Garmin *Swim*, Garmin International Inc, Olathe, KS. and Swimovate *PoolMatePro*, Swimovate Ltd, Middlesex, UK. [135,136,137,138]). Wrist-worn designs are a common feature and allow for user interaction with the devices (Figure 29). These systems all feature similar processing methods; data are stored on-board for immediate review or later downloaded to system specific software for analysis. It is seen that some of the general performance related variables such as stroke count and stroke rate found in research studies are also key features of commercially available products (Table 7).

The Garmin, FINIS and Swimovate products are geared towards a single user who wishes to gather useful performance related information when no coach is available. They would appear to be well suited to the task, especially for recreational swimmers, with their wrist worn design and interface. *AvidaMetrics* offers the potential to monitor activity of up to 25 athletes at one time, which is certainly attractive for gathering large scale training information and is more suited to competitive swim training. *AvidaMetrics* is the also the only commercially available system that featured a measure of lower limb activity. This system incorporates five sensors, two which are worn on the swimmers ankles, allowing this information to be gathered.

Certainly there is a growing interest in the commercialization of sensor based methods of analysing swimming performance, as evident from the number of patent applications that have emerged in recent years [23,24,32,36,38,39,42,49,65,67,88,90,91]. Unfortunately, no published research material is currently available that investigates the accuracy, reliability or validity of these products. Additionally, only limited information regarding the feature detection algorithms is available for these devices. Future research is warranted to fully assess the merits/demerits of these systems and their applicability for real-world settings.

**Table 7:** Details of system functionality provided by commercially available swimming sensor devices. The features described are similar to those described in research studies for the analysis of swimming performance.

Measured Parameter	AvidaSports *AvidaMetrics*	FINIS *Swimsense*	Garmin *Swim*	Swimovate *PoolMatePro*
Time	•	•	•	•
Stroke identification	•	•	•	
Stroke count	•	•	•	•
Stroke rate	•	•	•	
Split times	•	•	•	
Distance per stroke	•	•		
Breakout	•			
Average speed	•	•	•	•
Kick count	•			
Kick rate	•			
Lap counter		•	•	•
Efficiency				•
Intervals		•	•	
Distance		•	•	•
Calories		•	•	•

### 4.5. Sensor Attachment Locations

In selecting a sensor attachment location, it is important to have regard to the potential effects of that location on the desired measure of interest and on the quality of movement [139], as different measures are possible using different locations. Although the method of attachment is often unreported, attachment solutions include taping or strapping [62,70,83], wrist-watch style designs [12,15,32] or sensors incorporated into swim wear clothing [60,64]. Sensor movement may be inevitable and result in measurement inconsistencies, affecting the ability of sensor algorithms to accurately measure body motion [139]. This has implications for how sensors are attached to body segments.

For research purposes, it seems reasonable to use taping or a flexible medical plaster to attach sensors to body segments, ensuring accurate positioning that can be individually adjusted to suit a subject’s physique. In applied settings however a more convenient approach may be desired to ensure minimal set-up delay whilst also not significantly interfering with stroke mechanics. This is a key advantage of a wrist-watch styled approach; hence its popularity in commercially oriented monitors [32,38,91].

Unfortunately only a small number of papers discuss the relationship between comfort and sensor location or make attempts at quantifying the magnitude of measurement error introduced by sensor movement. An early prototype swim sensor described in 2008 by Davey, Anderson and James [11] was attached to the lower back using a belt but swimmer feedback indicated that it was unsuitable and caused excessive movement, especially during tumble turns. Bächlin and Tröster [62] used multiple sensors and aimed to minimise the risk of sensor slippage by using a belt with elastic stretch bands, Velcro fasteners and additional harnesses for individual sizing. It is unclear if this approach was successful or otherwise. A custom designed swimming suit with the sensor located inside a sealed pocket offers an interesting alternative attachment solution [64].

Participants in this study included a mixture of male and female, elite and recreational swimmers (N = 30), with a diverse range of body size and stature reported. However, it was unclear if the same suit was used for all subjects. Such an approach would clearly affect the exact location on the sacrum that the sensor was located [64]. Moreover, whilst no negative drag as a result of the suit was reported, no objective measure of this was provided and importantly the majority of subjects were recreational swimmers who may not adequately perceive drag effects. A variety of housing solutions have also been discussed. Clearly the main feature is that the device is watertight, and a variety of rubberised or plastic casings have been used. However, as much of the published work is based on prototype designs, this area remains underdeveloped, with many housing options lacking consideration for drag effects. Prototype designs may be bulky by nature and the intention of this work has been on algorithm development so it is not appropriate to be critical of such designs. However these clearly will impact on performance and it is a valid consideration for future development work.

#### 4.5.1. Upper Limb Locations

Swimming is an upper body dominant activity, with the majority of propulsion derived from the action of the upper limbs and the phases of arm movement result in changes in the acceleration of the entire body [1]. Therefore, in many of the reviewed studies, the authors chose to select locations on the arm, forearm or wrist [12,15,52,53,56,62]. This location has been particularly useful in studies investigating the various acceleration patterns exhibited by different swimmers. However, the use of a single device on the arm has some limitations which must be considered. For example, it has been found that wrist worn devices do not appear to be as accurate as sensors positioned on the torso for stroke type identification. Moreover, as consistent coordination between left and right arms or upper and lower limb actions cannot be guaranteed, the positioning of a sensor on one limb will not give a full and accurate picture of actual activity. Several studies have objectively demonstrated that variations in inter-arm coordination exist in swimming owing to various factors including swimming speed [99,140]; arm dominance [141]; physical disability [142]; energy cost [4]; exercise intensity [143] and skill level [140]. Furthermore, a similar variance exists between the coordination and synchronisation of the arms and legs for all swimming strokes [100,144]. All of these factors have implications for the accuracy of feature detection algorithms when using wrist mounted devices.

#### 4.5.2. Torso Locations

To investigate overall body motion a torso location provides a sensible alternative to the wrist. The back offers a practical solution towards balancing comfort with function, potentially minimizing the effect of drag and is found in a number of published studies [11,53,57,62,64,70,71,83]. As the sensor is located in close proximity to the body mass centre, a lower back location can detect whole-body accelerations and provide a good indication for overall swimming parameters such as mean velocity [64]; stroke type detection [11] or stroke rate analysis [11]. The sacrum is most frequently chosen, resulting in minimal intrusion both to stroke mechanics and the effects of body roll on the acceleration direction [53,56,64,70,71,83]. Back worn sensors are not well suited to a thorough kinematic analysis of upper or lower limb activity. A recent attempt was made to measure inter-arm stroke dynamics using acceleration and angular velocity recorded at the sacrum [70]. However, arm symmetry depends on many other variables other than just temporal characteristics, such as propulsive forces and the angular kinematics of the wrist, elbow and shoulder joints [1]. Recently, chest mounted sensors were described which demonstrated the benefits of back worn devices for monitoring whole-body motion, whilst also potentially allowing for integration of physiological data by incorporating an ECG (electrocardiograph) sensor [49,93].

#### 4.5.3. Head Locations

Locating a sensor on the head has many advantages. Similar to back worn devices, measures of overall body motion can be readily determined at the head. Furthermore, a head mounted device will not affect drag to the same degree as other body locations and the issue of attachment can be overcome by using a swim cap or goggle strap, which can be tightly fixed and is unlikely to result in excessive movement. As a consequence of these potential advantages, several of the reviewed studies have followed this approach to measure a wide range of parameters [35,44,52,78,86,89]. A possible concern could be that head movements or individual breathing styles may affect the output and make this location unsuitable, specifically for assessment of frontcrawl and backstroke as the head should remain relatively still. Another potential disadvantage of the head location is that motion of the head has six degrees of freedom, which may result in difficulty when extracting specific position or orientation based information, especially in developing swimmers who often struggle to maintain a static head positioning.

#### 4.5.4. Multiple Sensor Locations

Whilst the majority of systems described utilise a single sensor setup, it is a logical progression in the development of the technology to combine measurements from multiple sensors located at two or more body segments. Multiple sensor configurations have been used successfully for other human motion tracking [145] and sports applications [146,147]. Methods of handling large volumes of multi-sensor athlete data have also been described [148]. The potential benefit of a whole body system for biomechanical analysis in swimming include increased functionality over other described systems, allowing for a more detailed and thorough kinematic analysis of performance. For example, it has previously been suggested that the action of the legs can alter the trajectory of the wrist underwater, effectively improving the propulsive action of the arm, specifically by increasing stroke length and forward arm motion and also reducing backward movement in the sagittal plane [149,150]. Additionally, using multiple sensors allows for joint angular kinematical analysis to be carried out [94,95]. However, there is a trade-off that must be considered, as increasing the number of sensors will lead to increased drag, swimmer discomfort, altered swim mechanics and more complex signal processing and data transmission [53].

Swimming speed depends on maximising propulsive forces whilst also minimising resistive drag forces [1]. Elite swimmers routinely remove body hair and devote much attention to improving their streamlining. Body worn sensors may negatively influence drag and potentially hinder stroke dynamics. Additionally, active and passive drag may result in sensor artefact [53], potentially affecting algorithm accuracy, and should influence design decisions. However this important concern has been largely ignored by researchers. No study has yet objectively investigated the effects of drag due to body-worn systems, although some have reported subjective perceptions [33,57,62,64] and made attempt at low profile enclosures [53,62,83]. This issue will become increasingly significant as the move towards multiple sensor systems continues.

### 4.6. Technical Specifications of Inertial Sensor Designs Used in Swimming

A range of components have been incorporated into inertial sensor designs. Most common is an accelerometer [11,12,15,16,31,48,52,62,79,82,93], but gyroscopes are also found, typically when used in combination [33,51,53,56,57,64,70,71,83,94]. Acceleration has generally been measured along three axes for kinematic investigations, whereas gyroscopic information has been variously collected along either one [33,51], two [57,71] or three axes [53,56,64,70,83]. It was found that system designs have evolved from early models featuring uni-axial accelerometers to more recent devices where tri-axial accelerometers and tri-axial gyroscopes are now typical [10,53,83,87]. The inclusion of a magnetometer is also becoming more prevalent [60,90,91,99], whilst a recent study validated the use of a combined GPS and accelerometer device for kinematic analysis of swimming [73]. Integration of these sensors has also been attempted for physical activity monitoring [151,152] and in other sports [153,154], but the necessity to perform analyses in an outdoor environment limits functionality. Additionally whilst a magnetometer may increase the accuracy of the signal from the accelerometer and gyroscope, whose signals tend to drift, pool-operating machinery may hinder the magnetometer output [9].

**Figure 30:**
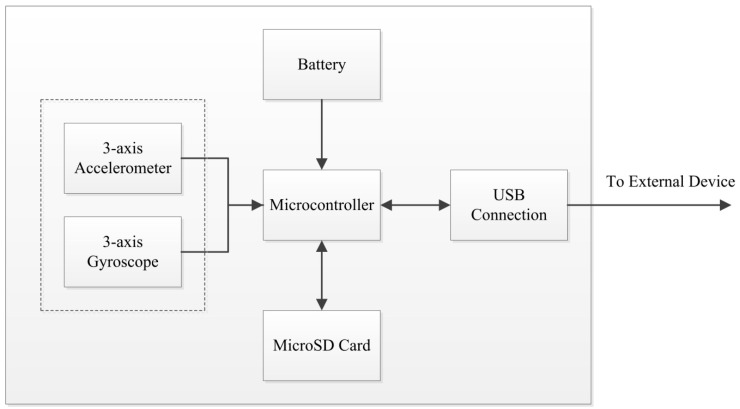
Example of typical system architecture found in inertial-sensor based devices used for the analysis of swimming.

Figure 30 provides an example of a typical system architecture which is emerging as a reference design for these systems and is reflective of the most commonly described systems in the literature. Many of the systems described are prototype systems that have been developed specifically for use in swimming research [53,74,83]. Additionally, various commercially available sensor devices such as Physilog (BioAGM, Switzerland) [64]; FreeSense (Sensorize, Italy) [47]; Minimax X (Catapult Sports, Australia) [46,86] and Shimmer (Shimmer, Ireland) [78,94] have also been used. These platforms are not specifically designed for use in swimming, therefore various modifications to make them suitable for use in aquatic environments have been developed, specifically to provide waterproofing solutions.

The selection of components should be dependent upon the desired output variable or the specific algorithm employed [139]. Whilst certain stroke mechanics may be analysed using only acceleration data [15,62], orientation information may also be required for analysing other skills, such as turns, for example [56]. The raw signal generated must undergo processing procedures to allow interpretation and analysis. Typically post-processing is conducted following data download to an external computer but recently on-board, real-time data processing has been described [57,71].

#### 4.6.1. Measurement Range

An essential feature of any sensor is that it provides an accurate measurement of the frequency and amplitude of human movement. Therefore, knowing these ranges for a given activity is important and will inform the sensor selection process. Human movement is in general considered to be at the lower end of the range of possible accelerations, with values of between −0.3 g to 0.8 g (−2.94 m∙s^−2^ to 7.85 m∙s^−2^) reported for walking and between 0.8 g to 4.0 g (7.85 m∙s^−2^ to 39.24 m∙s^−2^) for running [155]. Human body acceleration due to swimming falls between these activities, with values less than 2 g (19.62 m∙s^−2^) typical [57]. The measurement range of accelerometers reported in reviewed studies appears to cover this range appropriately, although agreement has not been reached on an optimum range and outliers can be found also [12,15,52]. The range of the gyroscope sensors varies between 8.7 and 26.2 rad·s^−1^, where reported. Measurement range may be influenced by sensor location, with more distally attached sensors requiring a greater range [139]. This is typical of gait analysis studies, whereby trunk worn devices have smaller ranges than those worn on the lower limbs [156,157]. However in the swimming studies reviewed it appears that this recommendation is not followed, with no consistency between the range selected and the attachment location whilst studies involving multiple sensors had a fixed range [53,62].

#### 4.6.2. Sampling Frequency

There appears to be little consensus in the extant literature as to the optimal sampling rate to record swimming variables, with a wide range of sampling frequencies described. This disparity may be due to sensor locations of selected studies; however a lack of justification for sampling rates chosen is evident from the literature. Very high sampling rates have the benefit of increased reproduction fidelity but increase computational power, storage capability and energy demands. In some instances, higher rates may be required to extract specific movement characteristics [158]. The Nyquist Sampling Theorem states that the recording frequency should be at least twice bandwidth of the signal being recorded. Early studies suggested that the lowest sampling frequency advisable for the accurate recognition of human motion was 20 Hz [159,160] although higher frequencies could be expected during limb movements [161]. By down-sampling accelerometer data originally sampled at 150 Hz, researchers have attempted to reduce the complexity of signal processing algorithms [162]. Although lower sampling rates achieved similar results in some cases, in general the lowest frequency (15 Hz) performed worst and accuracy and resolution decreased along with sampling frequency [162].

#### 4.6.3. Signal Filtering

Signal filtering processes are required as the signal to noise ratio can be low in a swimming setting [57]. Spectral analysis has revealed that a power peak frequency of approximately 6 Hz represents the movement of the arms and legs during a complete stroke cycle [163] and that frequencies in excess of 10 Hz are insignificant [164]. Butterworth and Hamming window filters are both commonly used in the extant literature. Butterworth filters provide a very flat frequency response in the passband and a key advantage over alternatives is that they do not require strict tolerances, unlike Chebyshev or Bessel filters [165]. Butterworth filters are also commonly used in other human movement related studies [122,166,167]. Some considered using a Chebyshev filter [57] but instead opted for a low pass Butterworth filter to avoid ripple voltages in the passband. A cut-off frequency of 2 Hz was applied to frontcrawl and backstroke, but this was deemed to smooth the data excessively for other strokes so higher frequencies (6 Hz for breaststroke; 8 Hz for butterfly) were chosen [57]. A Fourier filter has been shown to be accurate and effective for determining three dimensional orientations from a gyroscope in walking studies [168]. This method was also proposed for usage in swimming and one study followed this approach [95].

A common theme in the literature is that low pass filtering is conducted as the first stage of signal processing to remove unwanted noise components. However, it is important to note that there is a potential for valuable data to be lost if inappropriate filtering is adopted. Researchers should be aware of this fact and careful consideration should be given to the cut-off frequency employed as there is not a “one size fits all” solution to handling the raw data input. For example signals recorded from the back would have a much lower usable frequency content than those recorded from the arm and thus different cut-off frequency values would be used in a low-pass filter employed in these two cases.

#### 4.6.4. Data Storage and Transfer

Advances in data storage technology allows for increasingly compact solutions, offering capacities that are more than sufficient for recording swimming data in training environments. A 1 GB microSD card will allow for over 200 h of recording at 100 Hz [53]. For real-time systems, on-board storage is still required due to the volume of raw signal generated. One study incorporated 4 MB storage buffer, facilitating real-time implementation of data processing algorithms [57]. Interestingly though, raw acceleration signal was also transmitted along with the processed data, as both may be of relevance when a coach or sport scientist analyses performance. Real-time feedback is an exciting new area of research and will further enhance the standing of inertial sensor based systems within coaching communities. Rapid feedback on performance is vital to skill acquisition and has been found to improve technical performance in swimming [169].

However, the range of transmission is quite low, less than two meters in one study using Radio Frequency (RF) [53] and just 0.7 m for an optical wireless link when operated in turbulent water [52], thus feedback can only be provided to the swimmer and not the coach. This setup may be appropriate for recreational swimming analysis but is unsuited to elite swimming environments. This is a limitation of the majority of the data transmission options described. One paper did report a tested RF transmission range of 35 m at 0.25 m water depth, but unfortunately without providing additional methodological details [57].

#### 4.6.5. Power Supply

Power consumption of wearable sensor devices is an on-going area of investigation within the research community and as multiple sensor designs become more commonplace; so too will the requirement for balancing power consumption to avoid overload [170]. It has been suggested that the main constraint on the size and mass of MEMS systems is the power source; highlighting the requirement for low power signal processing methods [162]. Eight hours of battery life can be achieved using a high density lithium polymer cell incorporating sleep states and variable clock rates [53]. One system is capable of 48 h of continuous recording across multiple sensors using a 250 mAh 3.7 V rechargeable battery [62]. Lithium ion batteries are not without limitations for use in aquatic environments due to the fire risk associated with damage or leakages. An alternative solution may include super-capacitors or carbon-nanotube based energy stores. Another potential lies in energy harvesting in the surrounding electromagnetic environment; but further research is required in these areas [170,171,172,173,174].

## 5. Conclusions

This paper aimed to provide a systematic and critical review of inertial sensor use within swimming, focusing on methods that have been described for extracting key performance related variables for different phases of swimming and the consequences of different sensor attachment locations. Of the 87 papers included in this review, 62 of them (71.3%) have been published since 2010. Consequently, this field of study is relatively new and rapidly expanding. The development of this technology has advanced from early prototype models capable of simple stroke recognition to more recent systems that have provided for temporal; kinematic; kinetic and physiological analyses. Systems have been described that are capable of analysing starts; turns and free swimming parameters for a range of swimming strokes.

Much of the work has focused on extracting variables using relatively simple processing techniques, such as peak detection and zero-crossing. This requires an understanding of the features of the raw acceleration and angular velocity signals and their relevance to swimming performance as well as an appreciation for individual differences in stroke mechanics. Detecting other variables require more complex solutions. The accurate determination of swimming velocity, for example, is a current area of much research, with different methods being explored including integration and regression techniques. It remains to be seen which process will prove to be most appropriate. This is perhaps expected for a growing field of research but such inconsistency will undoubtedly result in confusion amongst coaches and sports scientists and also makes comparisons between studies difficult. It is important that best practice approaches to analysing swimming performance using inertial sensors are developed to ensure a greater adoption of the technology in applied settings and increased confidence in the accuracy of specific designs. Perhaps the greatest challenge at present when considering algorithm development is ensuring that the systems can robustly handle the individual movement characteristics of different swimmers and with high accuracy. It could be argued that the research community as a whole needs to move beyond low level signal processing techniques such as peak detection and to move towards more complex signal processing and data analysis techniques in order to achieve solutions to these ongoing issues and to provide a greater depth of analysis potential to swimming coaches and practitioners.

It has also been found that many different sensor locations have been used to date. Advantages of choosing a single site include ease of use and reduced cost but with limitations on the depth of analysis possible. Moreover, many algorithms described are specific to the location chosen and once selected should not be used interchangeably [139]. Multiple sensors mounted on various body segments offer increased analytical potential as reflected in recent studies. Certainly, the selection of an appropriate location or locations must be related to the measurement variable of interest due to the specific mechanics and coordination patterns of the four competitive strokes. The same function, such as stroke count, cannot always be best measured for different strokes using the same location.

As would be expected in a new area of research, there remains a large number or directions for future work to exploit. The variety of system specifications described is vast but with little consideration for the potentially negative effects of drag owing to their design. The accuracy of some feature detection algorithms may be questioned, such as those for lap time and stroke count. There remains a need for more thorough validation of systems and processes as much work to date has involved low participant numbers and insufficient detail regarding validation procedures that have been carried out. The lack of statistical analysis performed in some of these studies to determine the significance of the findings is also a concern. For example, Siirtola, Laurinen, Roning and Kinnunen [59] reported accuracy levels of greater than 99% for their stroke count algorithm but did not provide any statistical analysis and details of the method of validating the sensor data were not properly reported.

Several aspects of swimming analysis are largely unexplored but are vital from a coaching point of view. These include increasing the array of variables that can be measured, not just for free-swimming but also for the analysis of starts and turns which remains underdeveloped. Joint angular kinematics has not received sufficient research attention and to date no study has attempted to describe the action of the shoulder joint, which is paramount in swimming. Developing the kinetic potential of sensor based technology would open up a new avenue for many coaches.

Future work also needs to focus on applied studies to demonstrate how this technology can be used to influence coaching practice. The work of Fulton and colleagues [33,34,51] into kicking patterns is important as they are utilising sensor-based technology to optimise performance in an elite coaching setting. Similar examples are lacking in the research literature. Future applied research investigating other swimming strokes and involving elite able-bodied swimmers as participants are warranted, in order to convince the coaching population that sensor have a place in swimming training. Currently, the awareness and usage of sensor-based technology in applied swimming programmes is very low [8].

Commercial systems appear to be more geared for recreational swimmer and lack sufficient depth of analytical potential, as well as operational validity, to be of relevance currently in elite swimming. Additionally, research should look to include all four competitive strokes when validating feature detection algorithms in order to increase the applicability of this technology for real-world settings.

The evidence presented to date would suggest that inertial sensor technology has enormous potential to influence swim coaching practice in the coming years. Due to the difficulty in obtaining accurate data in aquatic environments, there is a strong demand for sophisticated analysis tools to quantify key performance related variables such as acceleration and velocity. MEMS based technology has the potential to deliver the required accuracy, precision and speed of feedback. Ultimately, however, this technology is competing against video-based analytical tools and researchers should continue to strive towards providing sufficient evidential basis of the merits of inertial sensors. Until such time, it is likely that coaches will continue to rely on traditional approaches for the analysis of swimming performance.
